# Stimulus-Responsive DNA Hydrogel Biosensors for Food Safety Detection

**DOI:** 10.3390/bios13030320

**Published:** 2023-02-24

**Authors:** Huiyuan Wang, Xinyu Wang, Keqiang Lai, Juan Yan

**Affiliations:** Laboratory of Quality and Safety Risk Assessment for Aquatic Products on Storage and Preservation (Shanghai), Ministry of Agriculture, Shanghai Engineering Research Center of Aquatic-Product Process & Preservation, College of Food Science and Technology, Shanghai Ocean University, Shanghai 201306, China

**Keywords:** smart DNA hydrogel, DNA nanomaterials, aptamer, nucleic acid amplification techniques, food contaminants, food safety monitoring

## Abstract

Food safety has always been a major global challenge to human health and the effective detection of harmful substances in food can reduce the risk to human health. However, the food industry has been plagued by a lack of effective and sensitive safety monitoring methods due to the tension between the cost and effectiveness of monitoring. DNA-based hydrogels combine the advantages of biocompatibility, programmability, the molecular recognition of DNA molecules, and the hydrophilicity of hydrogels, making them a hotspot in the research field of new nanomaterials. The stimulus response property greatly broadens the function and application range of DNA hydrogel. In recent years, DNA hydrogels based on stimulus-responsive mechanisms have been widely applied in the field of biosensing for the detection of a variety of target substances, including various food contaminants. In this review, we describe the recent advances in the preparation of stimuli-responsive DNA hydrogels, highlighting the progress of its application in food safety detection. Finally, we also discuss the challenges and future application of stimulus-responsive DNA hydrogels.

## 1. Introduction

Hydrogels are materials with a three-dimensional (3D) network structure formed by chemical or physical cross-linking and have been widely used in the field of biomaterials due to their unique physical properties such as good hydrophilicity, softness and elastic consistency [1,2,3]. Deoxyribonucleic acid (DNA) is the central molecule of life, with excellent biocompatibility, stability, precise recognition and high versatility [4,5,6]. DNA hydrogels are highly cross-linked porous nanomaterials, which are web-like structures formed by cross-linking DNA molecules and are one of the most important representatives of macroscopic 3D DNA materials [7]. DNA strands can be flexibly manipulated to construct highly predictable and structured DNA networks since they are programmable, complementary, and chemically alterable [8]. In addition, the 3D scaffold of the DNA hydrogel provides mechanical rigidity and has multiple binding sites. The combination of the unique biological function of DNA molecules and the good skeletal function of hydrogel makes DNA hydrogel suitable for wide applications in biosensing [9], drug delivery [10], environmental protection [11], food safety monitoring, and making new medical devices [12].

Stimulus-responsive DNA hydrogels have gained more attention as a particular type of DNA hydrogel with the smart property of being able to respond to the environmental stimuli [13]. Based on Watson–Crick base pairing between conventional DNA strands or the characteristic response behavior of functional nucleic acids (such as i-motif structures and G-quadruplexes), stimulus-responsive DNA hydrogels undergo changes in phase, volume, or other physicochemical properties triggered by temperature, pH, light, metal ions, biomolecules or other external factors [14,15]. In fact, stimulus-responsive DNA hydrogels can be divided into different categories according to different principles. The response factor in DNA hydrogel may be a non-nucleic acid [16,17,18] or a nucleic acid sequence, such as an aptamer [19,20,21]. In addition, according to different stimuli factors in the environment, they can be divided into two types: chemical triggering and physical triggering hydrogel [22]. Additionally, according to the number of environmental stimuli factors, they can be divided into single-stimulus and multi-stimulus-responsive DNA hydrogels [23]. Stimulus-responsive DNA hydrogels offer more possibilities for the application of intelligent materials in biology due to their gel ↔ sol–gel phase change properties [24,25].

According to the World Health Organization (WHO), adequate access to safe and nutritious food is essential for sustaining life and promoting health [26]. However, food is frequently contaminated with bacteria, viruses, fungi, and parasites, which cause diseases such as hemolytic uremic syndrome, irritable bowel syndrome, and Guillain-Barre syndrome [27]. In addition, with the spread of modern agriculture and the rapid industrialization of food, the risk of residues of pesticides, veterinary drugs and illegal additives in food is increasing [28]. Environmental and water pollution can also have an impact on food safety. Therefore, effective and sensitive food safety monitoring methods are particularly important. Many attempts have been made, but the existing methods still have certain shortcomings. Traditional methods such as culture and colony counting are less efficient and sensitive [29]. Other methods such as enzyme-linked immunoassay (ELISA), polymerase chain reaction (PCR) and high-performance liquid chromatography (HPLC), despite having improved sensitivity, are still not suitable for on-site detection or field use due to the complexity and poor portability of the instruments [30,31]. New monitoring methods need to be developed in the food industry to cope with the severe food quality and safety issues.

**Figure 1:**
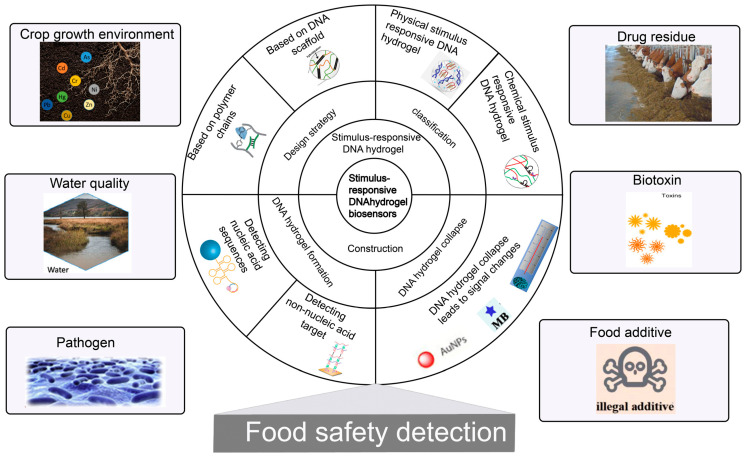
A schematic representation of the content reviewed in this work [32,36,37,38].

A biosensor is an analytic device that combines a biological component with a physicochemical detector to detect analytes [32]. Compared to existing food safety detection methods, biosensors offer significant advantages such as low cost, rapid analysis, and ease of use [33,34]. Due to the advantages of biocompatibility, programmability and good external response, stimulus-responsive DNA hydrogel can be used as a recognition element for biosensors to improve the sensitivity and specificity [35]. In this review, we will first outline general strategies for constructing stimulus-responsive DNA hydrogels. Then, according to different external stimuli, two types of DNA hydrogels are introduced: physical and chemical stimuli-responsive DNA hydrogels are introduced. Finally, the latest research progress on stimulus-responsive DNA hydrogel-based biosensors for food safety detection, challenges and an outlook of the future developments of this rapidly evolving field are presented, respectively (Figure 1).

## 2. Preparation and Classification of Stimulus-Responsive DNA Hydrogel

### 2.1. Design Strategies of Stimulus-Responsive DNA Hydrogels

At present, there are two main strategies for synthesizing stimulus-responsive DNA hydrogels. One is DNA strands as a hydrogel scaffold. It involves a change in the DNA scaffold leading to a phase change in the hydrogel. The other is to use other polymer chains as the scaffolds which are modified with functional DNA sequences. This section describes the characteristics of those two preparation strategies.

#### 2.1.1. DNA Hydrogel Based on DNA Scaffold

This strategy is generally applicable to pure DNA hydrogels, which are mainly realized by DNA self-assembly or enzymatic ligation [22]. Self-assembling of DNA nanostructures [39] and nucleic acid amplification techniques [40,41] can be used to form DNA scaffolds with stimulus-responsive components added to the backbone as the joint part. The most commonly used responsive components are aptamers for target substances [42], pH-responsive i-motif structures [43] and T-A·T or C-G·C^+^ triplexes [44], metal-ion-responsive DNAzymes [14], C-Ag^+^-C and T-Hg^+^-T metal-bridged double helix DNA [45], G-quadruplex structures [46], light-responsive azo-benzene intercalated DNA structures [47], and responsive nanoparticles such as ZnO nanoparticles.

**Figure 2:**
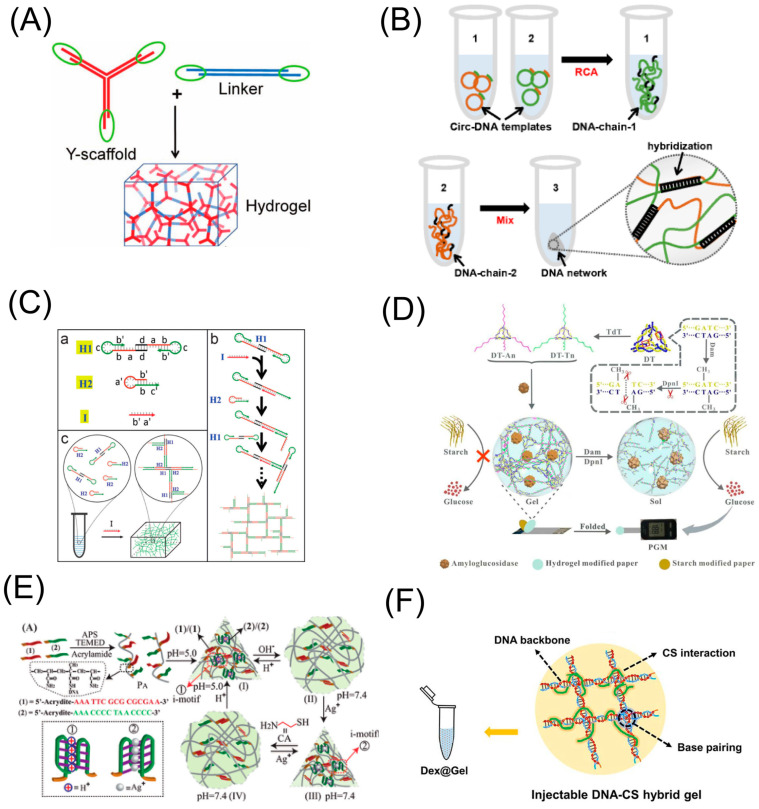
Different strategies prepared for DNA hydrogel. (**A**) pH-responsive DNA hydrogels prepared on the basis of Y-type DNA nanostructures. Copyright (2011) Wiley [19]. (**B**) DNA hydrogels with DNA backbone prepared by double RCA strategy. Copyright (2020) ACS publications [48]. (**C**) HCR-based preparation of DNA hydrogels for DNA scaffolds. Copyright (2017) Wiley [40]. (**D**) DNA hydrogels formed from DNA scaffolds prepared by TdT elongation. Copyright (2020) ACS publications [49]. (**E**) pH-responsive DNA hydrogels prepared on the basis of acrylamide chains. Copyright (2016) Wiley [50]. (**F**) DNA-CS hydrogels prepared on the basis of electrostatic adsorption. Copyright (2021) Elsevier [51].

The preparation of DNA hydrogels based on DNA scaffolds was initially carried out by the ligation or DNA self-assembly. For example, a common approach is assembling DNA scaffolds from various branched-DNA architectures (X-, Y-, or T-shaped DNA) with complementary sticky ends. In 2006, Luo’s team [39] formed DNA hydrogels using three separate DNA building blocks and T_4_ ligase mediation. This strategy involves inter-hybridization and ligation of DNA building blocks with T_4_ ligase to form DNA hydrogels. This method is characterized by the ability to adjust the mechanical properties of the DNA hydrogel by adjusting the type and concentration of the DNA building blocks to suit the actual requirements. However, the enzyme-mediated cross-linking is time-consuming, complex and costly. Researchers have started to design linkers to enable self-assembly between DNA building blocks. Liu’s team [19] used Y-type DNA nanostructures as scaffolds and linear double-stranded DNA as linkers to form a DNA hydrogel (Figure 2A). The sticky ends of the Y-type nanostructures and the linker are complementary to each other which leads to hydrogel formation. Moreover, their other work is simpler in design. Only a kind of Y-DNA nanostructure units was used as the backbone, whose i-motif domain contains two cytosine-rich stretches. When the pH changes to acidic, the cytosines in the C-rich domains become partially protonated, which leads to the formation of a C-CH^+^ triple hydrogen bond, thereby forming a pH-responsive DNA hydrogel [20]. Compared to other preparation methods, the strategy based on the DNA self-assembly does not require complex steps, but its high cost is still a fact that cannot be ignored.

In addition to DNA nanostructures, nucleic acid amplification method is another common strategy for the formation of a DNA scaffold. Among them, rolling circle amplification (RCA), an isothermal DNA replication technique, has been proven very useful for the mass production of DNA scaffolds [52]. It generates large amounts of ultralong ssDNA with periodic patterns [53], which can be physically entangled to build DNA hydrogel networks. Alternatively, repeated functional nucleic acid fragments can be obtained by the design of a specific circular template. Additionally, then, DNA hydrogels can be fabricated through the chemical linkage between functional nucleic acid fragments. Tian’s team used RCA method to produce long DNA chains containing pH-sensitive i-motif repeat sequences [54]. The results showed that the DNA hydrogel has good stability and efficient pH response and has the potential for the study of pH-stimulated drug release. An ingenious design of double RCA strategy has also been developed [48]. Two ssDNA chains produced by RCA were used to construct DNA hydrogel through the hybridization between complementary sequences (Figure 2B). The two main advantages of using RCA to prepare DNA hydrogels are the simplicity and the efficiency. It does not require precise temperature control and can reach more than 200 cycles in only 1 h. However, the low mechanical properties of DNA hydrogels prepared using RCA may limit their application in some special or complex environments.

Hybridization chain reaction (HCR) is another isothermal amplification reaction that constructs DNA scaffolds without the involvement of enzymes [55]. It has the advantage of being able to quantitatively regulate the generation of DNA hydrogels by designing the DNA sequence and reaction concentration. In addition, this method does not require template design and can provide hydrogels with better mechanical properties than that by RCA. Wang et al. designed a stimulus-responsive DNA hydrogel based on the clamped HCR method. In the work, two identical hairpin strands, H_1_, form a hairpin dimer, and upon the addition of an initiator, the hairpin dimer opens on one side and binds to the other hairpin strand, H_2_, thereby triggering the transition of the hydrogel from a sol to a gel [40] (Figure 2C). Similar to the process of self-assembly DNA nano units, this method requires fixed sequences and high concentrations of DNA monomers, which may be detrimental to the functionalization and application of DNA hydrogels.

Terminal deoxynucleotidyl transferase (TdT)-mediated amplification is capable of catalyzing the polymerization of deoxyribonucleotide extensions of DNA molecules at the 3′-OH ends of double- or single-stranded DNA without the need for a template. Complementary hybridization of the poly-A or poly-T tails of the building blocks formed by the TdT technique enables the formation of DNA hydrogels [49] (Figure 2D). The introduction of TdT into DNA scaffolds formation reduces the initial DNA concentration required and gives DNA hydrogels strong mechanical properties.

Pure DNA hydrogel has excellent biocompatibility and biodegradability. However, regarding other properties, it is worth noting that the microstructure of pure DNA hydrogels prepared by the above different methods are not exactly the same. For example, the microstructure of hydrogels formed through RCA is mainly a nanoflower structure. Generally, RCA-based DNA hydrogels are structurally loose and have low mechanical properties. In contrast, HCR-based DNA hydrogels are mostly cross-linked by hydrogen-bonds, showing a porous three-dimensional microstructure. This improves the stability and mechanical properties of the DNA hydrogel. Similar to HCR-based hydrogels, TdT-based hydrogel has a highly porous microstructure, which is also a preparation method favorable for robust hydrogels.

#### 2.1.2. DNA Hydrogel Based on Other Polymer Chain

The former strategy mainly targets pure DNA hydrogels, but pure DNA hydrogels are suffering from high cost and low mechanical properties. Therefore, there has always been an interest in developing DNA-polymer hybrid hydrogels.

DNA-polymer hybrid hydrogels are usually prepared by grafting DNA onto polymer chains and linking the polymer chains through DNA hybridization or supramolecular interactions [56]. Attachment of DNA to the polymer chains is usually achieved in two ways. First, DNA can be modified with acrylic acid and a DNA copolymer can be synthesized with acrylamide by copolymerization. In addition, the formation of amide or disulfide bonds, Michael addition reactions and electrostatic interactions also contribute to the post- modification and attachment of DNA to the native polymer chains [57].

Compared with DNA hydrogels based on DNA scaffolds, the hydrogels prepared with polymer chains have stronger mechanical stability. However, the biocompatibility and degradability of the latter are relatively poor. Cheng et al. [21] prepared a DNA-acrylamide hydrogel for the detection of Chlamydia trachomatis. Two acrylic acid modified DNA strands were copolymerized with acrylamide to form two DNA-polymer strands, which were assembled by DNA hybridization to form a DNA hydrogel. Yu et al. [50] designed a DNA-acrylamide hydrogel in response to pH and Ag^+^, where different responsive DNA sequences were copolymerized with acrylamide and assembled to form a DNA hydrogel in response to external stimuli. (Figure 2E).

In addition to DNA copolymerization with acrylamide, DNA polymer chains can be formed by DNA post-modification with other hydrophilic polymers such as carboxymethyl cellulose (CMC) and chitosan (CS). Changing the ratio of DNA to polymer concentration can modulate the mechanical properties of the hydrogel. Wang et al. [58] designed a photo-responsive DNA-CMC based hydrogel. The amino-modified DNA was linked to CMC via amide bonds, and DNA hydrogels were formed by DNA hybridization. A DNA-CS hydrogel for drug delivery was prepared by Chen et al. [51]. This work does not require the modification of DNA and enables the attachment of DNA to the polymer by virtue of the electrostatic adsorption between DNA and chitosan. Moreover, the microstructure and mechanical properties of the hydrogel were modulated by adjusting the chitosan content (Figure 2F). Additionally, other stimulus-responsive DNA sequences, such as G-quadruplex and supramolecular structures (such as azobenzene), can be assembled onto polymer chains, rendering the resulting DNA-polymer hydrogels stimulus-reactive.

In Table 1, these two approaches to construct stimulus-responsive DNA hydrogel are summarized, which refer to different types of scaffolds and the construction strategies of scaffolds.

### 2.2. Classification of Stimulus-Responsive DNA Hydrogel

According to the different environmental stimuli, we divide DNA hydrogels into two types: physical-stimulus-responsive and chemical-stimulus-responsive DNA hydrogels. Physical-stimulus-responsive DNA hydrogels mainly refer to the phase change of hydrogels triggered by physical factors such as pH, temperature, light, heat, electrical fields, magnetic fields, ultrasound irradiation and sonication. In contrast, chemical-stimulus-responsive DNA hydrogels are stimulated by chemical substances such as metal ions, biomolecules (DNA, enzymes, proteins and small molecules), redox, enzymes and solvents. In this section, an overview of common physical and chemical-stimulus-responsive DNA hydrogel assembly methods and their working principles are presented.

#### 2.2.1. Physical-Stimulus-Responsive DNA Hydrogel

##### pH-Responsive DNA Hydrogels

pH is an indicator of the concentration of hydrogen ions in a solution. When the pH in a solution is less than 7, the solution is dominated by H^+^ and its concentration is greater than that of OH^-^. Some DNA structures are sensitive to changes in pH, resulting in conformational changes. For example, the partial protonation of the C-rich sequence makes i-motif structure very sensitive to pH changes. At pH 5, the partially protonated cytosine (CH^+^) interacts with the unprotonated cytosine via hydrogen bond to form a stable parallel double helix. At pH 8, acid-base neutralization causes the structure to deconvolve into a single chain [59,60]. T-A·T and C-G·C^+^ triple helix structures are also highly sensitive to pH changes. At pH 7, the T-rich sequence and the T-A sequence form a T-A·T structure through hydrogen bonding. At pH 10, the T-A·T structure is dissociated due to the deprotonation of thymine residues. Similar to i-motif structure, the protonated C-G·C^+^ forms a triple helix structure under acidic conditions at pH 5, while the neutral conditions at pH 7 promote the dissociation of the structure [44]. The introduction of those pH-responsive DNA structures into DNA hydrogels induces phase transitions under environmental pH stimulation.

**Figure 3:**
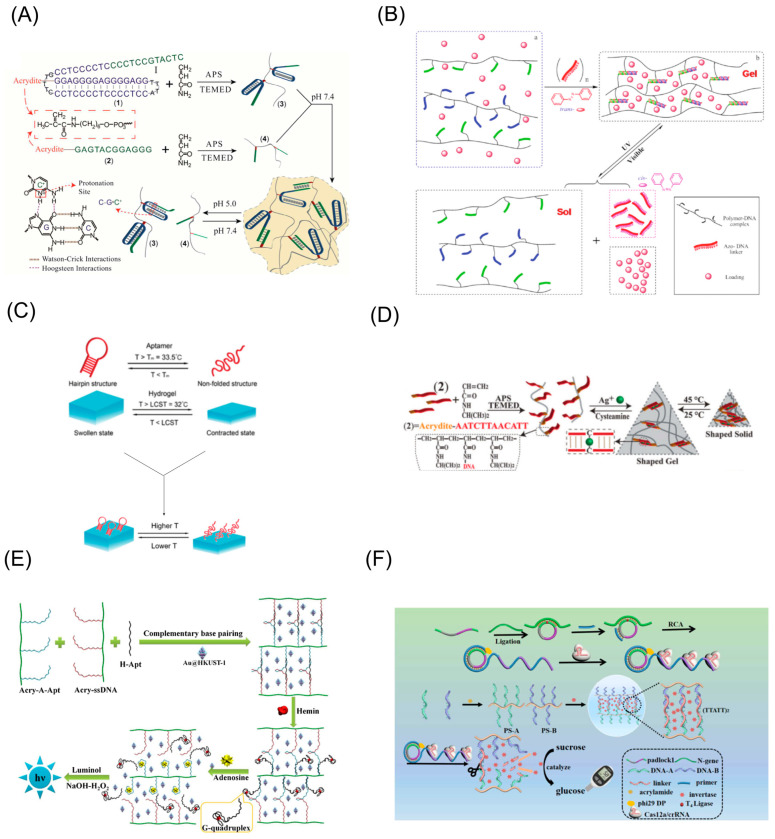
Schematic diagram of chemical and physically stimulus-responsive DNA hydrogels. (**A**) Schematic Illustration for acid-resistant and physiological pH-responsive DNA hydrogel preparation and insulin release. Copyright (2012) RSC publications [61]. (**B**) Azo-incorporated DNA linker can cross-link the DNA-polymer conjugates and form the hydrogel. Copyright (2010) ACS publications [62]. (**C**) Schematic diagram of the reversible response of aptamer-functionalized hydrogels to temperature. Copyright (2021) RSC publications [63]. (**D**) Schematic diagram of a DNA hydrogel that responds to Ag^+^. Copyright (2014) Wiley [64]. (**E**) Biomolecule-responsive DNA hydrogels based on aptamer response formation. Copyright (2019) Elsevier [65]. (**F**) Schematic representation of the DNA hydrogel sensing platform for sensing N-gene via a PGM. Copyright (2022) Elsevier [66].

Xu et al. [54] designed a pH-responsive DNA hydrogel that uses RCA to periodically align i-motif structural sequences in DNA strands. The RCA reaction generates thousands of i-motif fragments, which can easily form i-motif cross-linked structures between chains under acidic conditions, thus forming DNA hydrogels. Ying’s team [61] designed an acid-tolerant stimuli-responsive DNA hydrogel with cross-linked structures A-motif and i-motif that both remained stable under acidic conditions. Due to the fact that the A-motif structure was stable at pH 1.2~3 and the C-motif structure was stable at pH 4~6, leaving the acidic environment would lead to the breakage of the DNA hydrogel. Based on this property, DNA hydrogels have been used as drug delivery vehicles. The C-G·C^+^ structure and the T-A·T structure were combined to produce a pH-responsive DNA hydrogel with dual memory (Figure 3A). At pH 5, the formation of the C-G·C^+^ triple helix structure caused the DNA hydrogel to be in a liquid state. At pH 7, the formation of the T-A·T structure and the dissociation of the C-G·C^+^ structure transformed it into a gel state. At pH 10, the dissociation of the T-A·T structure caused it to change again to a liquid state. This study makes it possible to release the drug molecules from hydrogels as a carrier in different pH environments [44].

##### Light-Responsive DNA Hydrogel

Common photosensitive substances applied to DNA hydrogels generally include azobenzene (Azo), dithienylethene (DTE) and *o*-nitrobenzyl ester. Azo contains two aromatic rings linked by a nitrogen-nitrogen double bonds and realizes the reversible transformation from a trans-isomer (stable) to cis-isomer (loose) under different light irradiation. In addition, it has attracted much attention in the fields of materials, biological probes and optical information storage due to its excellent properties such as good light resistance, fast optical response and ultra-high storage density. Azo has also been used in the preparation of DNA hydrogels as a crosslinking component, allowing the hydrogel to trigger changes in mechanical properties and phase transitions under light stimulation [67]. Kang et al. [62] prepared a light-responsive DNA hydrogel as a drug delivery vehicle (Figure 3B). Under UV light irradiation, Azo shifted to the cis conformation causing its structure to break up while under visible light; its structure was restored.

As a representative of the pericyclic reaction, DTE has excellent fatigue resistance and thermal stability, and is one of the most popular photosensitive substances available in optical storage, molecular switching, and fluorescent imaging. Under the irradiation of UV light, its conformation undergoes a closed-loop change, while under visible light, its conformation is reduced to an open-loop body. Based on this principle, a number of works have been developed using DTE as a crosslinking structure for light-responsive DNA hydrogels [68]. The design provides the possibility of controlling the structure and properties of innovative materials in the future.

The *o*-nitrobenzyl compounds are one of the most studied photosensitive substances. Due to their well-known photolytic mechanisms and tunable chemical structures, many materials introduce them into cross-linked structures. Under light irradiation, *o*-nitrobenzyl undergoes an energy change to give the final photolysis product. Based on such photolytic principles, Willner’s team [69] synthesized DNA hydrogel membranes that can change patterns using *o*-nitrobenzyl phosphate groups. The pattern and mechanical properties of this hydrogel membrane were modulated by stimulation with light.

##### Temperature-Responsive DNA Hydrogel

DNA is a thermosensitive material capable of reversible denaturation (or melting) in response to temperature stimuli. Therefore, DNA hydrogels based on reversible phase transition of DNA duplexes can be prepared by using the reaction environment of heating-cooling cyclic [70]. Heat-induced cleavage of pure DNA hydrogels is related to the melting temperature of the DNA, i.e., the T_m_ value, which depends on the number of G-C base pairs in the DNA strands. In 2018, Zhu’s team designed a thermosensitive DNA hydrogel for cellulase recycling [71]. When the ambient temperature is heated to 55 °C, the DNA hydrogel transforms into a liquid. When the ambient temperature was cooled to 4 °C, the DNA hydrogel returned to the gel state and encapsulated the cellulase. Additionally, for DNA-polymer hybrid hydrogels, based on the selection of a lower critical solution temperature (LCST) polymer or an upper critical solution temperature (UCST) polymer as a hydrogel skeleton, the thermosensitive properties of the hybrid hydrogels can be controlled by both the thermosensitive properties of the polymers themselves and the T_m_ values of the DNA strands. For example, poly-N-isopropylacrylamide (PNIPAAm) provides a strategy for fabricating tunable temperature-active hydrogels. Zhang et al. designed a DNA hydrogel incorporating PNIPAAm for the capture and release of immunoaffinity cells [63]. When the temperature rose to the common critical temperature of the thermosensitive material and the aptamer, the material contracted and the aptamer denatured to release the cells, enabling a temperature-controlled capture to release the cells (Figure 3C).

#### 2.2.2. Chemical-Stimulus-Responsive DNA Hydrogels

##### Metal-Ion-Responsive DNA Hydrogel

Some specific DNA sequences are sensitive to metal ions, such as the two metal-bridged structures C-Ag^+^-C, T-Hg^+^-T and G-quadruplex. In general, C-C base mismatch cannot promote the normal hybridization of the nucleic acid strands, but the presence of Ag^+^ in the system can make it specifically bind to the C base to form a C-Ag^+^-C structure. Similarly, when Hg^+^ is present in the system, it can bind specifically to the T-T base pair [72,73]. The presence of K^+^ in the environment induces the formation of G-quadruplexes from G base-rich sequences, while the removal of K^+^ leads to the dissociation of G-quadruplexes [74]. These three DNA structures are widely used in metal-ion-responsive DNA hydrogel preparation. Willner’s team developed an Ag^+^-responsive DNA hydrogel (Figure 3D). The strategy uses Ag^+^ to stimulate the formation of a C-Ag^+^-C DNA double helix structure and eliminates Ag^+^ by adding cysteine to cause DNA hydrogel breakage [64]. Kahn et al. [75] designed a DNA hydrogel containing G-quadruplexes. The hydrogel was based on hairpin DNA structures modified on acrylamide chains forming a cross-linked structure via the HCR reaction. The mechanical property of the DNA hydrogel was modulated by adding or eliminating K^+^ in the environment, resulting in a conformational change in the G-quadruplex. DNAzyme is a catalytic DNA molecule screened in vitro, which can specifically catalyze the cleavage of substrate sequences under the stimulation of metal ions. Therefore, the addition of DNAzyme to the substrate sequence enables DNA hydrogels to acquire metal responsiveness [76]. Stimulated by Zn^2+^, the DNAzyme cleaves the substrate sequence, and the cross-linked structure is disrupted, leading to the release of cancer cells encapsulated in the hydrogel.

##### Biomolecules-Responsive DNA Hydrogel

With the rapid development of DNA nanotechnology, aptamers, as a class of oligonucleotides that can specifically bind to biological molecules (enzymes [41], DNA [77], proteins, biological small molecules, etc.), are being used more widely. Compared to antibodies, aptamers have more stability, more accessibility, are easily chemically synthesized and modified, and high affinity and specificity to their targets. In the preparation of DNA hydrogels, aptamers are generally designed as crosslink components. In the presence of target biomolecules, competitive binding of the aptamer to the biomolecules can result in a phase transition of the DNA hydrogel [78]. An ATP-responsive DNA hydrogel was developed based on adenosine triphosphate (ATP) aptamers [79]. In the presence of ATP, the volume of the DNA hydrogel showed a significant reduction, suggesting that the specific binding of aptamer to ATP altered the cross-linked structure of the DNA hydrogel. In 2019, Luo’s team [65] designed a dual molecule-responsive DNA hydrogel. ATP aptamer and hemin aptamer formed a sandwich DNA structure with ssDNA and produced DNA network. When hemin and adenosine were added, the specific recognition of the molecules and their aptamers caused the rupture of the hydrogel (Figure 3E).

Restriction nucleases can cleave DNA sequences with specific sites. For example, Cas12a nuclease, when activated by crRNA, is capable of non-specifically cleaving long-stranded DNA at specific binding sites. Ma et al. [66] designed a Cas12a/crRNA enzyme-responsive DNA hydrogel for the detection of specific genes. In the presence of target gene, Cas12a enzyme activity was activated and led to the disruption of the cross-links, thereby releasing the invertase, which converted sucrose to glucose for signal readout with a glucose meter (Figure 3F).

The characteristics of DNA hydrogels in response to some specific physical and chemical stimuli and common stimulators are systematically summarized in Table 2.

## 3. Stimulus-Responsive DNA Hydrogel-Based Biosensor

Biosensors are devices that are created by combining a sensitive transducer element with a selective biorecognition element [80]. Stimulus-responsive DNA hydrogel can be a reliable material for the biological characteristic elements in biosensors due to their specificity and rapid response to stimuli. Currently, there are two main approaches to design biosensors based on stimulus-responsive DNA hydrogels. The first one is based on the principle of DNA hydrogel collapse, which is generally achieved by releasing signal molecules (or various enzymes) encapsulated in the hydrogels or triggering direct signal changes through gel dissolution. The second one is based on the mechanism of DNA hydrogel formation, in which external stimuli lead to the construction of DNA hydrogel, and in this process, the signal changes. In this section, we will briefly introduce the strategies for constructing stimulus-responsive DNA hydrogel biosensors based on these two principles.

### 3.1. DNA Hydrogel Collapse Principle

Stimulus-responsive DNA hydrogels collapse upon a particular stimulus, where the hydrogel-encapsulated signal molecules or active enzymes flow out, thereby changing the color, fluorescence, or Raman signal of the solution.

**Figure 4:**
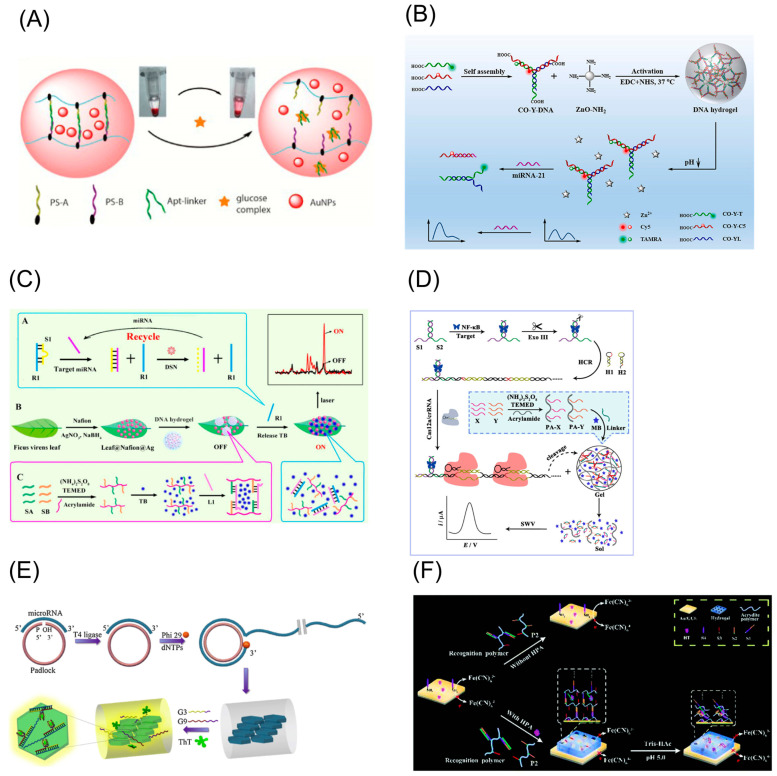
Main strategies for stimulus-responsive DNA hydrogel biosensors. (**A**) Working principle of DNA hydrogel containing embedded AuNPs for visual detection of glucose. Copyright (2013) RSC publications [16]. (**B**) Schematic diagram of a stimulus-responsive DNA hydrogel fluorescent biosensor for the detection of miRNA 21. Copyright (2022) Elsevier [17]. (**C**) Working principle of DNA hydrogel encapsulating TBs for detection of miRNA 15. Copyright (2017) RSC publications [78]. (**D**) Schematic diagram of an electrochemical NF-κB p50 detection strategy. Copyright (2022) Elsevier [93]. (**E**) Illustration of label-free polygonal-plate fluorescent-hydrogel (PPFH) biosensor based on the RCA for miR-21 quantification. Copyright (2020) Elsevier [94]. (**F**) Schematic diagram of a DNA hydrogel electrochemical biosensor for the detection of HPA. Copyright (2017) RSC publications [95].

Colorimetry is a method of qualitative or quantitative analysis by using the absorption characteristics of colored substances in the system to specific wavelength light. Colored substances may be originally present in the system or may be generated by certain reactions [81,82]. Some metal nanoparticles show different colors because of their different sizes and morphologies, such as gold nanoparticles (AuNPs) [83] and silver nanoparticles (AgNPs) [84]. The color changes can also be achieved using the catalysis of oxidoreductase, such as horseradish peroxidase (HRP) [85] and Glucose oxidase (GOD) [86]. For example, HRP catalyzed the oxidation of TMB with the assistant of H_2_O_2_ for the colorimetric assay. The other is nanomaterials (or nanostructures) with enzyme-like catalytic effects, namely nanoenzymes [87], such as nanometals and nanometal oxides (such as Fe_3_O_4_) [88], carbon-based nanomaterial [89] (such as carbon nanotube, graphene and its derivatives) and porous organic frameworks [90,91] (such as metal–organic frameworks, MOFs; and covalent-organic frameworks, COFs). In a strategy in which a glucose-responsive DNA hydrogel colorimetric biosensor was used to visually detect glucose, when glucose was added, the gel collapsed and AuNPs encapsulated in the hydrogel were released. A distinct red color was observed in the supernatant, which was determined by UV-Vis spectrophotometry [16] (Figure 4A). In addition, the color change also can be achieved by reactive enzymes or nanoparticles. PtNPs/Cu-TCPP (Fe) was reported to have enzymatic activity, which can trigger TMB reaction and lead to an increase in absorbance value in the work of DNA hydrogel colorimetric biosensor for the detection of creatine kinase MB [92].

The fluorescence method has several advantages, including quick response, ease of use, and non-invasiveness [96]. It is a qualitative and quantitative analysis of substances by means of signal changes in the output of fluorescent signal molecules [97]. The main fluorescent signal molecules commonly used today are the traditional organic fluorescent dyes and the emerging fluorescent nanoparticles. Traditional organic fluorescent dyes such as fluorescein and rhodamine have the advantage of being easily labelled and accessible and are, therefore, ripe for commercial labelling [98]. However, they also have disadvantages such as easy photobleaching, low fluorescence intensity and weak fluorescence lifetime [99,100]. Emerging fluorescent nanoparticles such as quantum dots (QDs) have superior optical properties, but currently may suffer from difficulties in synthesis and high surface activity [101]. DNA hydrogel biosensors incorporated with the fluorescence strategy are now being widely used. In a stimulus-responsive DNA hydrogel fluorescent biosensor strategy for the detection of MicroRNA(miRNA) 21 [17], fluorescent signal molecules TAMRA and Cy5 modified on the DNA strand are released when a target is present, resulting in a change in the fluorescence ratio (Figure 4B). In addition, a stimulus-responsive DNA hydrogel biosensor combined with QDs was used for the detection of miRNA 141 [102]. The DNA hydrogel was stimulated by miRNA 141 to rupture, releasing QDs, which led to an enhanced fluorescence signal.

The surface-enhanced Raman scattering (SERS) technique combines the advantages of high chemical specificity, high sensitivity and surface selectivity [103]. Moreover, the SERS assay is fast, reusable and simple to operate [104]. Therefore, the sensing strategy of DNA hydrogel SERS biosensors has received increasing attention [105]. The design key of the assay is the change in the number of Raman signal molecules or the regulation of the distance between the Raman reporter molecules and the active substrate when the hydrogel collapses. In a stimulus-responsive DNA hydrogel SERS biosensor for the detection of miRNA 155 [78], toluidine blue (TB) molecules were encapsulated in the DNA hydrogel and kept away from the Raman substrate. After the stimulation, the DNA hydrogel ruptured, causing TB molecules to release and approach the substrate, thus generating an enhanced Raman signal (Figure 4C).

In addition, a stimulus-responsive DNA hydrogel electrochemical biosensor is realized by encapsulating an electroactive substance in a DNA hydrogel and obtaining a good electric signal through the release of the electroactive substance after the stimulation. As shown in Figure 4D, the cross-linked strands of the DNA hydrogel were cleaved by Cas12a enzyme, releasing the encapsulated methylene blue (MB). Quantitative analysis of the target was achieved by monitoring the significantly enhanced electrical signal [93].

The various signal output methods mentioned above have their own characteristics. For example, the colorimetric method is simple and fast, but its sensitivity and stability need to be improved; the fluorescence, although sensitive and stable, requires large, complex instruments; the hand-held Raman spectrometer can be used for SERS detection, but this method requires a highly reproducible and stable active substrate or a highly sensitive SERS tag; and electrochemical methods are susceptible to external interference. Selecting an appropriate construction strategy of stimulus-responsive DNA hydrogel biosensor and the corresponding signal output and analysis methods will help to improve the detection performance of the hydrogel biosensor.

### 3.2. DNA Hydrogel Formation Principle

In contrast to the previous collapse principle of DNA hydrogel, the states of the signal molecules can also be changed and captured during the formation of the hydrogel under the external stimulus. At the same time, similar to the above principle, this kind of sensor system can still achieve the qualitative or quantitative analysis of target substances through colorimetry [106], fluorescence [107], Raman [108], electrochemistry [109] and so on.

Nucleic acid amplification techniques introduced above for preparing DNA hydrogel are typically used for the construction of such sensing strategies because these amplification techniques often require a nucleic acid primer strand to initiate an amplification reaction, such as RCA and HCR. Accordingly, if the target substance is a nucleic acid sequence (DNA or RNA) [94], the target itself can be designed as a primer for a nucleic acid amplification technique (Figure 4E). Through the capture and analysis of signal molecules encapsulated in the DNA hydrogel generated by the primer regulation, the quantitative detection of the target sequence can be achieved. If the target substance is a non-nucleic acid, the detection task of the target substance is often converted to the detection of the aptamer by adding a biological recognition process of the target substance and the aptamer [95] (Figure 4F).

Both strategies for constructing stimulus-responsive DNA hydrogel biosensors (collapse or formation of DNA hydrogels) essentially use the capture of a target to cause a phase change in the gel to achieve a change in state of the signal molecule for the purpose of analysis and detection. These mechanisms are summarized in Table 3. Comparatively speaking, those methods based on hydrogel collapse mechanism are more widely used because of their simplicity in the design. 

## 4. Stimulus-Responsive DNA Hydrogel Biosensors for Food Safety Detection

With the development of the modern food industry, food safety is not only threatened by traditional pathogens, heavy metals, fungi, pesticide and veterinary drug residues, but also by illegal food additives and the use of genetically modified (GM) food [110,111]. In addition, the soil environment and water quality are also closely related to food safety [112,113]. Although the traditional food safety monitoring methods such as high-performance liquid chromatography (HPLC), gas chromatography (GC), and mass spectrometry (MS) have been widely used and can effectively detect food contaminants, there are still some problems that cannot be ignored, such as complex instruments, low detection efficiency, needing well-trained professional and technical personnel and so on [114,115]. Biosensors are increasingly used in food safety monitoring due to their exceptional specificity and sensitivity, fast response time, ease of operation and low cost [116]. Currently, stimulus-responsive DNA hydrogel-based biosensors have aroused wide interest in the field of food safety monitoring and become a hot spot in this field [117,118]. In this section, according to different types of food safety problems, we will present a detailed review of the application of the smart DNA hydrogel-based biosensors in the field of food safety detection.

### 4.1. Heavy Metals

Heavy metals, which are metals with a density greater than 4.5 g/cm^3^, include gold, silver, copper, iron, mercury, lead and cadmium. They are very difficult to be biodegraded, but instead can be enriched thousands of times by the biomagnification of the food chain. When they finally enter the human body, they can interact strongly with proteins and enzymes, rendering them inactive, causing poisoning and serious damage to human health. The main possible sources of current heavy metals in food are as follows: (1) Crop soils: heavy metal contamination of crop soils has become a global problem, with 12 million tons of food contaminated with heavy metals each year in China alone [119]. (2) Untreated irrigation water: it can significantly alter soil quality, increase the levels of trace heavy metals in soil and crops, and be a source of impact on food quality and safety [120]. Goodman’s team [121] studied grain and vegetables irrigated with untreated sewage water in Tianjin, China, and found more serious heavy metal contamination in both grain and vegetables, with Cd, Cr, Pb, and As levels in wheat exceeding national limits. The concentrations of Cd, Pb, and As in vegetables were also higher than the federal safety limits. (3) The migration of food processing or packaging material.

**Figure 5:**
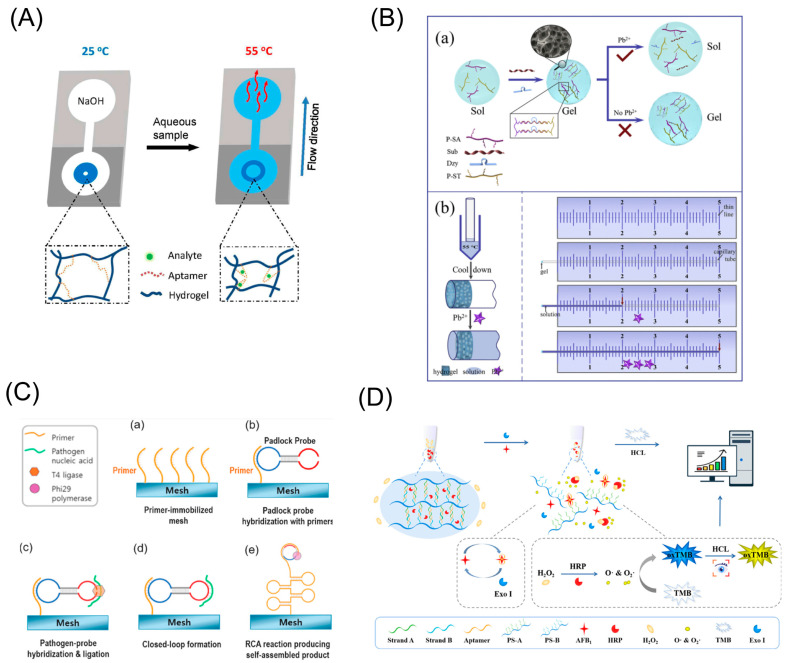
Schematic diagram of stimulus-responsive DNA hydrogel biosensors for food safety detection. (**A**) Schematic diagram of a stimulus-responsive DNA hydrogel thermal biosensor for the detection of Hg^2+^. Copyright (2015) RSC publications [124]. (**B**) Schematic illustration of the DNA-based hydrogel for the detection of Pb^2+^. Copyright (2020) Elsevier [18]. (**C**) Schematic diagram of a stimulus-responsive DNA hydrogel colorimetric biosensor for the detection of COVID-19. Copyright (2021) Elsevier [125]. (**D**) Detection principle of AFB_1_-responsive aptamer-functionalized DNA intelligent hydrogel biosensor. Copyright (2020) Elsevier [126].

When stimuli-responsive DNA hydrogel biosensors are applied to the detection of heavy metals, there are two hydrogel collapse modes can be used. (1) Heavy metals in the system compete with the aptamers, which are used as the linker of the hydrogel, leading to the hydrogel collapse [122]. (2) Heavy metals in the system activate DNAzymes, which cuts the DNA hydrogel and causes the gel to collapse [123]. For example, an aptamer-based stimulus-responsive DNA hydrogel microfluidic chip sensor was fabricated for the detection of Hg^2+^ [124]. Aptamer-specific binding of Hg^2+^ causes DNA hydrogel rupture. The increased chip flow rate altered the heat of NaOH dissolution (Figure 5A). Thermometer measurement of temperature increments enables quantitative analysis. The sensor does not require a signal molecule and the quantitative approach based on a forehead thermometer can reduce instrumentation the requirements for instruments and the difficulty of experiments. At the same time, its ability to be reused will help significantly reduce the cost of the assay. A good linear range of 0.1–10 μM and a detection limit of 0.081 μM will facilitate in situ detection of Hg^2+^. In addition, in a capillary sensor based on a DNAzyme cleaved DNA hydrogel strategy for the detection of Pb^2+^ [18], the presence of Pb^2+^ activated DNAzyme activity and cleaved DNA hydrogels, resulting in changes in capillary flow rate for qualitative and quantitative analysis through visual observation (Figure 5B). The sensor takes advantage of the excellent sensitivity and specificity of the DNAzyme without encapsulating nano-enzymes or signal molecules and can detect pb^2+^ as low as 10 nM at a distance of 20 mm within 1 h.

Methods based on these two detection modes have also been used for the detection of other heavy metals. In addition, with the rise of the nuclear power industry, the contamination of uranium in water needs to be taken seriously. Rapid and easy on-site detection methods for uranyl ions in water were developed. For example, Huang et al. [127] designed a DNA enzyme hydrogel biosensor based on UO_2_^2+^ response. The presence of UO_2_^2+^ was able to confer DNA enzyme activity, causing the DNA hydrogel to collapse and release AuNPs for colorimetric detection. The sensor is easy to use, with the detection limits as low as 37 nM, enabling highly sensitive in situ detection of UO_2_^2+^.

### 4.2. Pathogen Monitoring

Foodborne infections continue to be one of the leading causes of illness worldwide, according to the WHO [128]. In the United States, for example, more than 45 million people are affected by foodborne illness each year, resulting in over 120,000 hospitalizations, 3000 deaths, and an economic loss of USD 15 billion [129]. Unlike chemical contaminants, which are usually present at some stage of food production and are relatively easy to control, the impact of pathogens on food safety can occur at all stages. Effective pathogen surveillance tools can improve the early warning of possible microbial hazards at all stages of food production, from raw materials to commodities, and enable timely control measures to be taken. The plate culturing and counting method is the “gold standard” of conventional pathogen detection because it is widely applicable, inexpensive and accurate. However, plate culture is relatively time-consuming, typically taking two to four days [130]. The rapid polymerase chain reaction (PCR) detection time has been reduced to 20 min and has the advantages of high sensitivity and specificity [131]. However, the reaction is overly dependent on the temperature control system, which limits its application in field detection. Stimulus-responsive DNA hydrogel biosensors are able to respond to pathogenic hazards in each stage of food production in a short time without complex detection equipment, so it can better meet the requirements of field testing.

*Escherichia coli* (*E. coli*) is a common pathogenic bacterium in food, and Shiga toxin-producing *E. coli* (O157:H7) infections are one of the leading causes of foodborne illness [132]. These food pathogens can cause acute gastroenteritis, resulting in bloody stools [133]. Zhang et al. [134] designed a DNA hydrogel biosensor for the visual detection of *E. coli* O157:H7. The work was based on the DNA hydrogel formation principle. In the presence of *E. coli*, aptamers bound specifically to *E. coli* and released an initiator stand, which hybridized with circular template sequence and triggered an RCA reaction for hydrogel formation. As a result, qualitative and semi-quantitative analysis of *E. coli* can be achieved with the naked eye alone. The method successfully utilizes the DNA hydrogel biosensors to realize visual detection of *E. coli,* and 4 × 10^3^ CFU/mL of *E. coli* can be detected in less than 1 h.

*Vibrio parahaemolyticus* (V.P) is a major cause of diarrhea when eating seafood, and the number of infections and outbreaks cause by V.P is increasing. Yu et al. [135] designed a smart hydrogel biosensor for monitoring V.P. The aptamer specifically binds to ATP, causing the gel to rupture and release gold nanoclusters (AuNCs), enabling rapid qualitative and quantitative analysis of V.P by combining fluorescence signal visualization with microfluidic microarrays. The highlight of this method is the detection of its metabolic release, ATP, which is more readily accessible to the gel network, improving the efficiency and sensitivity of the assay and taking advantage of the specificity of the aptamer recognition, ultimately achieving a sensitivity as low as 10 CFU⋅mL^−1^ in a detection time of less than 1 h. In addition to *E. coli* and V.P mentioned above, Bacillus anthracis and Salmonella were also detected using smart hydrogel-based biosensors [136,137].

In addition to pathogenic bacteria, many foodborne viruses also pose a threat to food safety. Food-borne viruses are viruses that use food as a vehicle to cause disease in humans, such as avian influenza virus, mad cow disease virus, foot-and-mouth disease virus, rotavirus, hepatitis virus, adenovirus and norovirus [138]. The use of stimulus-responsive DNA hydrogel biosensing strategies for foodborne viruses has also recently attracted considerable interest [139,140]. For example, Li’s team designed a target-responsive DNA hydrogel fluorescent sensor to detect AIV H_5_N_1_ [141]. The DNA hydrogel biosensor was modified with QDs and their quencher. When the H_5_N_1_ virus was present, the specific reaction of the aptamer with the virus led to the disruption of the DNA hydrogel and the separation of QDs and quencher, allowing the fluorescence signal to be detected. The findings showed that the newly created hydrogel-based aptasensor could identify AIV H_5_N_1_ at a lower detection limit of 0.4 HAU in 30 min. The method combines the fluorescence burst of quantum dots as a signal readout with a phase change in the hydrogel. This label-free, simple and inexpensive fluorescent biosensor has great potential for the rapid detection of AIV H_5_N_1_ in the field.

Cases of COVID-19 first emerged in late 2019, and the infection has since spread worldwide and become a pandemic. SARS-CoV-2 has now been found to have the potential to survive on the surface of meat tissue for several days, and thus may be a potential route of virus transmission and impact on food safety [142]. Kim et al. [125] devised a method that enables the rapid detection of SARS-CoV-2 using DNA hydrogels and microfluidic chips (Figure 5C). The presence of the virus completed the assembly of a dumbbell-shaped template, triggering an RCA reaction and successful synthesis of the DNA hydrogel. DNA hydrogel blocked the chip, inhibiting the flow of fluid. The virus was finally analyzed successfully by measuring the flow rate of the fluid in the glass tube. The technique had the lowest limit of detection (LOD) to date and could detect SARS-CoV-2 with an excellent LOD (~3 aM in 15 min or 30 aM in 5 min). The most outstanding advantage of this method is it is fast and simple, and it can adapt to most detection situations. This biosensor has the potential to rapidly detect SARS-CoV-2 on food raw materials.

### 4.3. Drug Residues

The current unregulated use of veterinary drugs and pesticides has led to drug residues in food raw materials. Common veterinary drugs include enrofloxacin, chloramphenicol, kanamycin, tetracycline and so on. Common pesticides include organochlorine and organophosphorus pesticides, such as chlorpyrifos, chlordane and methomyl. Residues of pesticides and veterinary drugs pose a major risk to the human body, such as improving bacterial resistance, increasing the risk of cancer, causing serious neuronal damage and even central nervous system death [143].

Streptomycin is an aminoglycoside antibiotic commonly used in agriculture and animal husbandry [144]. Streptomycin residues are frequently found in agricultural and food products such as eggs, meat, and milk [145]. The Chinese Ministry of Agriculture has set maximum residue limits (MRL) of 20 and 500 µg/kg for streptomycin in honey and chicken, and the European Commission has set a limit of 200 µg/kg for it in milk [146,147]. A SERS biosensor based on a kind of smart DNA hydrogel was developed to enable the detection of streptomycin [148]. The sensing strategy is based on the collapse of the DNA hydrogel and combines for the first time the good stability of gold nanorods (AuNRs) as a Raman substrate for the SERS detection of streptomycin. It achieves highly sensitivity and specificity, with an LOD of 4.85 × 10^−3^ nM and a stable Raman signal with a linear range of 0.01–150 nM. It has been successfully tested in milk and honey samples and has the potential to monitor streptomycin in food samples in the field.

Kanamycin is another representative aminoglycoside antibiotic with a similar scope of application to streptomycin, both being used for anti-bacterial treatment in animals, but more effective against Staphylococcus aureus. Its abuse can lead to adverse effects such as drug allergy, hearing damage, respiratory failure, ototoxicity and nephrotoxicity, as well as other adverse effects on human health through the food chain [149,150]. The MRL of kanamycin in milk is currently 150 µg/kg in the European Union and 200 µg/kg in China. Chen et al. [108] reported a stimulus-responsive DNA hydrogel SERS biosensor for the detection of kanamycin. The sensing strategy is based on the principle of DNA hydrogel construction and consists of three components: the preparation of gap-containing nanoparticles (GCNPs) as SERS tags; the competitive recognition of aptamers with kanamycin and the release of primers; and the preparation of DNA hydrogels. The highlight of this work is the use of Raman signals generated by different amounts of GCNPs captured in DNA hydrogel to reflect the amount of kanamycin in the system. The biosensor has been successfully used to detect milk and honey with detection limits as low as 2.3 fM, offering the advantages of high sensitivity and ease of operation.

In addition to the above two methods, a stimulus-responsive DNA hydrogel fluorescent sensor was used for the detection of oxytetracycline [151]. The method is easy to prepare by physically mixing graphene sheets, the adenosine and the inducer, and avoids tedious modification process and the consumption of the hydrogel. The detection limit is 25 μg/L, and the linear range is 25–1000 μg/L. Additionally, a DNA hydrogel thermal sensor was used for organophosphorus pesticide detection. The method is based on an exothermic reaction triggered by the release of hydrogen peroxidase after hydrogel collapse, thereby achieving efficient quantitative analysis [152].

### 4.4. Biotoxins

Natural toxins, also known as biotoxins, are typically peptides, small non-proteinaceous molecules, or proteins derived from natural sources such as plants, microbes (bacteria, fungi, viruses, and protozoans), and animals [153]. Biotoxins have repeatedly been linked to rising cancer incidence and mortality rates in many countries. They have garnered a great deal of attention in regarding food safety and human health. According to a WHO survey, humans are most likely exposed to toxins through contaminated food and water, causing chronic or acute poisoning [154]. Toxin exposure over time can result in gene mutation, teratogenicity, and cancer. The main methods currently used to detect biotoxins are chromatographic techniques and immunoassays [155]. Additionally, stimulus-responsive DNA hydrogel biosensors are increasingly being used for biotoxin detection [30,156].

Common biotoxins include plant toxins, animal toxins, marine toxins, and microbial toxins. Among these, aflatoxin is a class of compound containing difuran and coumarin skeleton, which is produced by fungal strains such as *Aspergillus flavus*, *A. nomius*, and *A. parasiticus* [157]. Aflatoxin B_1_ (AFB_1_) is the most toxic due to its high carcinogenicity, mutagenicity, immunosuppression, and ability to cause liver damage. According to Chinese Food Hygiene Standard regulations, 20 µg/kg of AFB_1_ is permitted in corn, peanuts, and peanut oil. For AFB_1_ detection of DNA hydrogel biosensors, there are those using pt nanoparticles (ptNPs) catalyzed H_2_O_2_ decomposition methods [158], those using urease catalyzed urea methods [159] and those using HRP catalyzed TMB discoloration [126] (Figure 5D). All these methods are based on the specific binding of aptamers leading to hydrogel collapse, but the difference lies in the way the signal is converted and analyzed. In these methods for AFB_1_ detection, the lowest detection limit of 1.77 nM was achieved.

Ochratoxin A (OTA) is a secondary metabolite produced by several Aspergillus and Penicillium fungal species [160]. Nowadays, OTA has received special attention due to its immunosuppressive, teratogenic, and carcinogenic properties [161]. Liu designed a hydrogel collapse-based biosensor for OTA detection [162]. When the hydrogel collapses, Au@Pt core–shell nanoparticles (Au@PtNPs) are released to decompose hydrogen peroxide, so as to carry out efficient quantitative analysis by observing the change in liquid flow distance on the chip. This strategy has the advantages of low cost and simple operation and has great potential for rapid detection. In addition, a hydrogel-based strategy for triggering fluorescence intensity changes was reported, achieving high specificity and the stable detection of OTA [163]. In the work, binding of the aptamer to the target releases the primer and triggers the RCA, generating a fluorescent hydrogel and resulting in a change in fluorescence intensity. This work innovatively integrates competitive binding patterns of inducers, complementary sequences and targets into DNA hydrogels for food safety testing. The biosensor has been successfully applied to the determination of OTA in beer with a detection limit of 0.01 ng mL^−1^.

Zearalenone (ZEN) is a xenoestrogenic mycotoxin primarily produced by Fusarium species [164]. Because of its significant pathogenicity and widespread distribution in animal feed ingredients and agricultural food, ZEN has emerged as a global public health concern. A colorimetric sensor combining hydrogel collapse and MOFzyme catalysis has successfully achieved high specificity and sensitivity for the detection of ZEN [165]. As MOFzyme has good oxidase activity, the presence of ZEN will lead to its exposure and catalyze the oxidation of TMB. This work is the first appearance of a hydrogel-coated MOFzyme strategy in the field of biosensors and has been successfully applied to the quantitative detection of ZEN in maize and soybean.

Moreover, the most common fumonisin, fumonisin B_1_ (FB_1_), which is capable of disrupting sphingolipid metabolism, was also detected by a MOF hydrogel colorimetric aptasensor approach [166]. The sensor combines MOF oxidase activity with the superior specificity of DNA hydrogels, ultimately generating a colorimetric signal. The method has been successfully applied to the determination of FB1 in maize and wheat with a linear range of 0.05-100 ng mL^−1^ and a detection limit of 0.024 ng mL^−1^.

### 4.5. Food Additives

Food additives are one of the most critical tools in the modern food industry to ensure the quality and flavor of food. However, excessive use of food additives may be detrimental to the health of consumers. Many countries have strengthened the regulation of the use of food additives and have introduced many measures to prevent the abuse of food additives. According to laws, regulations and standards, there are usually two ways to evaluate illegal food additives or food additive abuse: ingredient identification and content determination. The former aims to examine whether additives are used illegally, while the latter seeks to determine whether additives are overused [167]. The main illegal substances currently added to food are formaldehyde, melamine, Sudan red and cocaine. Food additives that are often used in excess include benzoic acid, sorbic acid and sweetener. This section describes methods for the detection of these substances based on smart DNA hydrogel biosensing strategies.

Among the illegal additions, melamine (MEL) is a raw material used in the manufacture of plastics, coatings, and adhesives [168]. Accumulation of MEL in the body may damage the kidneys and genitourinary system of a person. Although it is an industrial raw material, MEL is often used illegally to increase the apparent protein content of dairy products and animal feed due to its high nitrogen content. Wang et al. [169] combined stimulus-responsive DNA hydrogels with microfluidic chips to design a colorimetric biosensor. The DNA hydrogel can block the liquid channel; however, when MEL is present, the hydrogel collapses and liquid flows into the detection zone, allowing quantitative analysis based on the color change in the released AuNPs. The method is based on a hydrogel collapse and AuNPs colorimetric strategy and does not require large instruments, resulting in the efficient on-site detection of MEL. In addition, the quantitative analysis combined with smart phones is a highlight of this work. With a detection range of 0.2–50 μM and detection limits as low as 37 nM, it has been successfully applied to detecting MEL in milk powder.

Another common illegal additive is cocaine. It is normally used as a narcotic in the medical field, and it has addictive properties. Some unscrupulous businesses illegally add cocaine to food for financial gain. At the same time, some sports nutrition foods are being added to cocaine for potency. In a strategy based on cocaine-induced hydrogel rupture, the release of ptNPs catalyzes the decomposition of H_2_O_2_ leading to gas pressure changes, using a handheld pressure meter for highly specific and sensitive quantitative analysis [170]. The biosensor has a detection limit of 0.12 µM, high specificity and short detection time. Additionally, Wen’s team combined capillaries and stimulus-responsive DNA hydrogels to successfully achieve the trace detection of cocaine (1.17 nM) [171]. In the study, the researchers cleverly prepared the hydrogel in capillaries, where cocaine would change the permeability of the gel, resulting in differences in the flow rate in capillaries. Compared with other work, this strategy does not require external equipment to quantify the target and has a broader prospect in the field detection of cocaine and food safety monitoring.

### 4.6. Other Food Contaminants

Bisphenol A (BPA) is an endocrine-disrupting chemical (EDC) that is commonly found in everyday household products and various types of packaging [172]. Long-term intake of BPA may lead to health problems such as diabetes and obesity, as well as an increased risk of cancer. The current limit for BPA leaching in food packaging in China is 0.05 mg/kg. Gao et al. [173] designed a sensor based on the strategy of aptamer-specific binding leading to DNA hydrogel rupture. This method is combined with low-frequency NMR techniques and utilizes the aggregation of Fe_3_O_4_ superparamagnetic iron oxide nanoparticles (SPIONs) to trigger signal changes so as to detect the content of Bisphenol A in the drinking water. This biosensor exhibits a reasonable specificity and a wide linear range, with a detection limit of 0.07 ng mL^−1^ and good recovery in the sample.

With the development and application of agricultural biotechnology, the safety of GM food has become a growing concern around the world. In the detection methods of GM food, the polymerase chain reaction (PCR) is a common technique to detect whether the genetic material contains inserted foreign genes. Additionally, many rapid biosensing methods have been developed, such as isothermal-amplification-based biosensors [174], portable immune biosensors [175], and functional nucleic-acids-based biosensors [176]. Gryadunov et al. [177] designed a DNA hydrogel chip for the detection of transgenic crops. The method is based on the hybridization of amplified DNA and oligonucleotide probes on the chip, trigger the change in fluorescence intensity, and realizes the detection of four GM foods. Although the application of a DNA hydrogel biosensor in the detection of GM food is still limited, stimulus-responsive DNA hydrogel biosensors are expected to be utilized in the field detection of GM foods to improve the specificity and sensitivity of detection in the future.

In this section, a variety of methods for creating stimulus-responsive DNA hydrogel biosensors for the detection of different food contaminants in food safety monitoring are described and summarized in Table 4. It is worth mentioning that only those cases using stimulus-responsive DNA hydrogel biosensor are present in Table 4, and in the future, the smart hydrogel biosensing strategies will be applied to the detection of other kind of food contaminants.

### 4.7. Interference Suppression Strategies

Last but not least, biosensors are exposed to complex detection environments where the matrix may affect detection efficiency and effectiveness [178,179]. Especially for food safety testing, it needs to face the interference of more complex substrates in multi-component food [180]. Therefore, the study of interference suppression strategies is very important for the application of DNA hydrogel biosensors in the field of food safety monitoring. Stimulus-responsive DNA hydrogel biosensors are programmable, and designed to respond to specific targets and to some extent avoid the interference of substrates in complex environments. In addition, DNA hydrogels have unique porous structure advantages, which can avoid the interference of macromolecules. Mao et al. [181] designed a colorimetric biosensor for the detection of bilirubin in serum. The presence of a large number of interfering substances in serum often interferes with the detection. It was found that small molecules can diffuse rapidly in DNA hydrogels, while large molecules cannot, which helps to improve the efficiency and effectiveness of sensors for bilirubin detection. As with food safety testing, the structural advantages of DNA hydrogels can also help biosensors withstand complex environmental conditions. Pi et al. [182] designed a DNA hydrogel biosensor resistant to environmental interference for the detection of Hg^2+^ in water. By immobilizing DNA in a polyacrylamide hydrogel, the effect of pH, naturally dissolved organic matter and metal ligands on the detection of Hg^2+^ can be largely avoided. In fact, there are few studies on the DNA hydrogel biosensors for the interference suppression strategy in food safety detection, which may be one of the reasons that affect the commercial application of DNA hydrogel biosensors. Based on the significance of this issue, the research on it will attract more attention in this field.

## 5. Conclusions and Perspective

In conclusion, this study comprehensively reviews the specific construction mechanisms of the stimulus-responsive DNA hydrogel biosensors, various response factors of the external environment, and applications in food safety detection. Compared to other biomaterials, these kinds of smart three-dimensional nanomaterials have obvious multiple advantages. First, the controllability and designability of DNA molecules make it possible customize different DNA hydrogel structures on demand. Second, DNA hydrogels are easy to hybridize with other non-nucleic acids due to the easy modification and labeling of DNA molecules, and the properties and functions of the hydrogels can be continuously controlled in this process. In addition, the good compatibility of the gel system with other systems provides convenience for the design of biological application strategies of hydrogels.

At the same time, there are still some issues that should not be overlooked: (1) Both pure and hybrid DNA hydrogels rely on the expensive commercial synthesis of nucleic acid fragments, especially the former. More abundant, inexpensive sources or routes, for example, natural foods or food waste, may be the possible approaches in the future. (2) Most of the stimulus-responsive DNA hydrogel biosensing strategies are highly dependent on aptamers, which can be met by continually updating more abundant and stable aptamer resources, supported by evolving and efficient aptamer screening technologies. (3) The interference suppression strategies of the complex food matrix need to be further developed. Moreover, the application mechanism of hydrogel is relatively single, most of which is realized by the phase change of hydrogel caused by gel collapse or construction. This may limit its scope of application.

With the development of related technologies, smart DNA hydrogel biosensors with better performance will be developed in the future. The multi-factor responsiveness of DNA hydrogel will be further improved, so as to be applied to the simultaneous high-throughput detection of food contaminants. In addition, it is envisioned that stimulus-responsive DNA hydrogels can be used in conjunction with more signal output systems or hand-held, portable, commercial instruments for multi-target and on-site food safety screening. DNA hydrogel biosensors will be faster, more stable and cheaper, and the commercial DNA hydrogel assay is expected to be used for detecting food contaminants in daily life. In summary, stimulus-responsive DNA hydrogel will be a powerful tool and a prospective promising platform for the simple, rapid and highly sensitive detection of contaminants in the field of public food safety monitoring.

## Figures and Tables

**Table 1:** Stimulus-responsive DNA hydrogel design strategies.

Construction Strategy	Formation of the Scaffold	Advantages	Disadvantages	Refs.
DNA hydrogels based on DNA scaffolds	Self-assembly of DNA building blocks	Adjustable mechanical properties, simple steps	High cost and high synthetic conditions	[19,20,39]
	Rolling circle amplification (RCA)	Simplicity, efficiency, low synthesis conditions	Low mechanical properties	[48,54]
	Hybridization chain reaction (HCR)	Adjustable mechanical properties, simple sequence design	Complicated process and high cost	[40]
	Terminal deoxynucleotidyl transferase (TdT)	Strong mechanical properties, low cost	-	[49]
DNA hydrogels based on other polymer chains	Polyacrylamide chains	Strong mechanical stability	Low biocompatibility and degradability	[21,50]
	Carboxymethyl cellulose (CMC)	Adjustable mechanical properties	Complex modification process	[58]
	Chitosan (CS)	Convenient DNA ligation	-	[51]

**Table 2:** Classification of stimulus-responsive DNA hydrogels.

Type of Stimulation	Response Factors	Stimuli Factors	Refs.
Physical-stimulus-responsive	i-motif structure	pH	[54]
	A-motif, C-motif structure	pH	[61]
	T-A·T, C-G·C^+^ structure	pH	[44]
	azobenzene	light	[58]
	DTE	light	[68]
	*o*-nitrobenzylphosphate ester	light	[69]
	DNA material	temperature	[71]
	PNIPAAm, aptamer	temperature	[63]
Chemical-stimulus-responsive	C-Ag^+^-C	Ag^+^	[64]
	G-quadruplexes	K^+^	[75]
	DNAzyme sequence	Zn^2+^	[76]
	aptamer	ATP	[79]
	aptamer	Adenosine	[65]
	Cas-12a response sequence	Cas-12a	[66]

**Table 3:** Construction of the Stimulus-responsive DNA hydrogel biosensor.

Construction Principle	Sensor Type	Characteristics	Refs.
DNA hydrogel collapse principle	Colorimetric	Encapsulated AuNPs, PtNPs/Cu-TCPP	[16,92]
	Fluorescent	Modified with TAMRA and Cy5, encapsulated QDs	[17,102]
	SERS	Encapsulated TB molecule	[78]
	Electrochemical	Encapsulated MB	[93]
DNA hydrogel construction principle	Detecting nucleic acid sequences	Trigger nucleic acid amplification	[94]
	Detecting non-nucleic acid targets	Recognition between aptamer and targets, and trigger nucleic acid amplification	[95]

**Table 4:** Stimulus-responsive DNA hydrogel biosensors for food safety monitoring.

Types of Monitoring	Target Analytes	Response Factor	Sensor Type	Construction Strategy	Food Sample	Analytical Performance	Refs.
Heavy metals	Pb^2+^	DNAzyme chain	Colorimetric	Gel collapse DNA fragment measurement	Water	Linear range: 0–500 nM LOD: 7.7 nM	[82]
	Hg^2+^	Aptamer	Thermal	Gel collapse NaOH exothermic	-	Linear range: 0.1–10 μM LOD: 0.081 µM	[124]
	UO_2_^2+^	DNAzyme chain	Colorimetric	Gel collapse Encapsulated AuNPs	Water	Linear range: 0–600 nM LOD: 37 nM	[127]
Pathogen	E. coli O157:H7	Aptamer	Visualization	Gel construction	Milk	LOD: 4 × 10^3^ CFU mL^−1^	[134]
	V.P	Aptamer	Colorimetric	Gel collapse Encapsulated AuNCs	Fish products	Linear range: 10–10^7^ CFU mL^−1^ LOD:10 CFU mL^−1^	[135]
	AIV H_5_N_1_	Aptamer	Fluorescent	Gel collapse Modified QDs and quencher	-	Linear range: 2^−1.2^–2^6^ HAU 20 µL^−1^ LOD: 0.4 HAU	[141]
	SARS-CoV-2	Padlock probe	Colorimetric	Gel construction	-	LOD: ~3 aM in 15 min or 30 aM in 5 min	[125]
Drug residue	Streptomycin	DNAzyme chain	SERS	Gel collapse Encapsulated 4-MB	Milk, honey	Linear range: 0.01–150 nM LOD: 4.85 × 10^−3^ nM	[148]
	Kanamycin	Aptamer	SERS	Gel construction Encapsulated GcNPs	Milk, honey	Linear range: 1 pg L^−1^–10 ng L^−1^ LOD: 2.3 fM	[108]
	Oxytetracycline	Aptamer	Fluorescent	Gel collapse	Water	Linear range: 25–1000 μg L^−1^ LOD: 25 μg L^−1^	[151]
	Organophosphate pesticides	Aptamer	Thermal	Gel collapse Encapsulated catalase	-	Linear range: 0.0001–10 ng mL^−1^ LOD: 0.032 pg mL^−1^	[152]
Biotoxins	AFB_1_	Aptamer	Colorimetric	Gel collapse Encapsulated ptNPs	Beer	Linear range: 0–60 nM LOD:1.77 nM	[158]
	AFB_1_	Aptamer	Colorimetric	Gel collapse Encapsulated urease	Peanut	Linear range: 0.2–20 µM LOD: 0.1 µM	[159]
	AFB_1_	Aptamer	Colorimetric	Gel collapse Encapsulated HRP	Peanut oil	Linear range: 0–500 nM LOD: 4.93 nM	[126]
	OTA	Aptamer	Colorimetric	Gel collapse Encapsulated Au@PtNPs	Beer	LOD: 11.1 nM	[162]
	OTA	Aptamer	Fluorescent	Gel construction Modified Cy3	Beer	Linear range: 0.05–100 ng mL^−1^ LOD: 0.01 ng mL^−1^	[163]
	ZEN	Aptamer	Colorimetric	Gel collapse Encapsulated MOFzyme	Corn and soybeans	Linear range: 0.001–200 ng mL^−1^ LOD: 0.8 pg mL^−1^	[165]
	FB_1_	Aptamer	Colorimetric	Gel collapse Encapsulated MOF	Maize and wheat	Linear range: 0.05–100 ng mL^−1^ LOD:0.024 ng mL^−1^	[166]
Food additive	Melamine	Aptamer	Colorimetric	Gel collapse Encapsulated AuNPs	Milk and infant milk powder	Linear range: 0.2–50 μM LOD: 37 nM	[169]
	Cocaine	Aptamer	Gas	Gel collapse Encapsulated PtNPs	-	LOD: 2.3 fM	[170]
	Cocaine	Aptamer	Colorimetric	Gel construction	-	Linear range: 10 nM–100 μM LOD: 1.17 nM	[171]
Other	BPA	Aptamer	LF-NMR	Gel collapse Encapsulated Fe_3_O_4_ SPIONs	Water	Linear range: 10^−2^–10^2^ ng mL^−1^ LOD: 0.07 ng mL^−1^	[173]
	GM food	-	Fluorescent	-	Soybean, maize, potato	LOD: 0.5%	[177]

## Data Availability

Not applicable.

## Abbreviations

AFB_1_	Aflatoxin B_1_
AgNPs	Silver nanoparticles
ATP	Adenosine triphosphate
AuNCs	Gold nanoclusters
AuNPs	Gold nanoparticles
AuNRs	Gold nanorods
Au@PtNPs	Au@Pt core–shell nanoparticles
Azo	Azobenzene
BPA	Bisphenol A
CMC	Carboxymethyl cellulose
COFs	Covalent-organic frameworks
CS	chitosan
DNA	Deoxyribonucleic acid
DTE	Dithienylethene
ELISA	Enzyme-linked immunoassay
*E. coli*	*Escherichia coli*
FB_1_	Fumonisin B_1_
GC	Gas chromatography
GcNPs	Gap-containing nanoparticles
GM	Genetically modified
GOD	Glucose oxidase
HCR	Hybridization chain reaction
HPLC	High-performance liquid chromatography
HRP	Horseradish peroxidase
LCST	Lower critical solution temperature
LOD	Lowest limit of detection
MB	Methylene blue
MEL	Melamine
MiRNA	MicroRNA
MOFs	Metal–organic frameworks
MRL	Maximum residue limits
MS	Mass spectrometry
OTA	Ochratoxin A
PCR	Polymerase chain reaction
PNIPAAm	Propylacrylamide
PtNPs	Ptnanoparticles
QDs	Quantum dots
RCA	Rolling circle amplification
SERS	Surface-enhanced Raman scattering
TB	Toluidine blue
TdT	Terminal deoxynucleotidyl transferase
UCST	Upper critical solution temperature
V.P	*Vibrio parahaemolyticus*
WHO	World Health Organization
ZEN	Zearalenone

## References

[1] Soares P.A., de Seixas J.R., Albuquerque P.B., Santos G.R., Mourao P.A., Barros W., Correia M.T., Carneiro-da-Cunha M.G. (2015). Development and characterization of a new hydrogel based on galactomannan and kappa-carrageenan. Carbohydr. Polym..

[2] Tavakoli J., Wang J., Chuah C., Tang Y. (2020). Natural-based Hydrogels: A Journey from Simple to Smart Networks for Medical Examination. Curr. Med. Chem..

[3] Wu S., Wu S., Zhang X., Feng T., Wu L. (2023). Chitosan-Based Hydrogels for Bioelectronic Sensing: Recent Advances and Applications in Biomedicine and Food Safety. Biosensors.

[4] Pardo Y.A., Yancey K.G., Rosenwasser D.S., Bassen D.M., Butcher J.T., Sabin J.E., Ma M., Hamada S., Luo D. (2022). Interfacing DNA hydrogels with ceramics for biofunctional architectural materials. Mater. Today.

[5] Xu P.F., Noh H., Lee J.H., Domaille D.W., Nakatsuka M.A., Goodwin A.P., Cha J.N. (2013). Imparting the unique properties of DNA into complex material architectures and functions. Mater. Today.

[6] Simon A.J., Walls-Smith L.T., Plaxco K.W. (2018). Exploiting the conformational-selection mechanism to control the response kinetics of a “smart” DNA hydrogel. Analyst.

[7] Wang D., Hu Y., Liu P.F., Luo D. (2017). Bioresponsive DNA Hydrogels: Beyond the Conventional Stimuli Responsiveness. Acc. Chem. Res..

[8] Mo F.L., Jiang K., Zhao D., Wang Y.Q., Song J., Tan W.H. (2021). DNA hydrogel-based gene editing and drug delivery systems. Adv. Drug Delivery Rev..

[9] Beyer A., Pollok S., Rudloff A., Cialla-May D., Weber K., Popp J. (2016). Fast-Track, One-Step E. coli Detection: A Miniaturized Hydrogel Array Permits Specific Direct PCR and DNA Hybridization while Amplification. Macromol. Biosci..

[10] Jiang H.L., Kim Y.K., Lee S.M., Park M.R., Kim E.M., Jin Y.M., Arote R., Jeong H.J., Song S.C., Cho M.H. (2010). Galactosylated chitosan-g-PEI/DNA complexes-loaded poly(organophosphazene) hydrogel as a hepatocyte targeting gene delivery system. Arch. Pharm. Res..

[11] Wang Y., Zhu Y., Hu Y., Zeng G., Zhang Y., Zhang C., Feng C. (2018). How to Construct DNA Hydrogels for Environmental Applications: Advanced Water Treatment and Environmental Analysis. Small.

[12] Li C., Faulkner-Jones A., Dun A.R., Jin J., Chen P., Xing Y., Yang Z., Li Z., Shu W., Liu D. (2015). Rapid formation of a supramolecular polypeptide-DNA hydrogel for in situ three-dimensional multilayer bioprinting. Angew. Chem. Int. Ed..

[13] Chen M., Wang Y., Zhang J., Peng Y., Li S., Han D., Ren S., Qin K., Li S., Gao Z. (2022). Stimuli-responsive DNA-based hydrogels for biosensing applications. J. Nanobiotechnol..

[14] Chu J., Chen C., Li X., Yu L., Li W., Cheng M., Tang W., Xiong Z. (2021). A responsive pure DNA hydrogel for label-free detection of lead ion. Anal. Chim. Acta.

[15] Li J., Mo L.T., Lu C.H., Fu T., Yang H.H., Tan W.H. (2016). Functional nucleic acid-based hydrogels for bioanalytical and biomedical applications. Chem. Soc. Rev..

[16] Ma Y., Mao Y., An Y., Tian T., Zhang H., Yan J., Zhu Z., Yang C.J. (2018). Target-responsive DNA hydrogel for non-enzymatic and visual detection of glucose. Analyst.

[17] Yao S., Xiang L., Wang L., Gong H., Chen F., Cai C. (2022). pH-responsive DNA hydrogels with ratiometric fluorescence for accurate detection of miRNA-21. Anal. Chim. Acta.

[18] Jiang C., Li Y., Wang H., Chen D., Wen Y. (2020). A portable visual capillary sensor based on functional DNA crosslinked hydrogel for point-of-care detection of lead ion. Sens. Actuators B.

[19] Xing Y., Cheng E., Yang Y., Chen P., Zhang T., Sun Y., Yang Z., Liu D. (2011). Self-assembled DNA hydrogels with designable thermal and enzymatic responsiveness. Adv. Mater..

[20] Cheng E., Xing Y., Chen P., Yang Y., Sun Y., Zhou D., Xu L., Fan Q., Liu D. (2009). A pH-triggered, fast-responding DNA hydrogel. Angew. Chem. Int. Ed..

[21] Cheng L., He Y., Yang Y., Chen J., He H., Liu Y., Lin Z., Hong G. (2022). Highly reproducible and sensitive electrochemical biosensor for Chlamydia trachomatis detection based on duplex-specific nuclease-assisted target-responsive DNA hydrogels and bovine serum albumin carrier platform. Anal. Chim. Acta.

[22] Iqbal S., Ahmed F., Xiong H. (2021). Responsive-DNA hydrogel based intelligent materials: Preparation and applications. Chem. Eng. J..

[23] Willner I. (2017). Stimuli-Controlled Hydrogels and Their Applications. Acc. Chem. Res..

[24] Vazquez-Gonzalez M., Willner I. (2020). Stimuli-Responsive Biomolecule-Based Hydrogels and Their Applications. Angew. Chem. Int. Ed..

[25] Wang C., Zhang J. (2022). Recent Advances in Stimuli-Responsive DNA-Based Hydrogels. ACS Appl. Bio Mater..

[26] Ferrari A.G., Crapnell R.D., Banks C.E. (2021). Electroanalytical Overview: Electrochemical Sensing Platforms for Food and Drink Safety. Biosensors.

[27] Saravanan A., Kumar P.S., Hemavathy R.V., Jeevanantham S., Kamalesh R., Sneha S., Yaashikaa P.R. (2020). Methods of detection of food-borne pathogens: A review. Environ. Chem. Lett..

[28] Shenashen M.A., Emran M.Y., El Sabagh A., Selim M.M., Elmarakbi A., El-Safty S.A. (2022). Progress in sensory devices of pesticides, pathogens, coronavirus, and chemical additives and hazards in food assessment: Food safety concerns. Prog. Mater Sci..

[29] Zhang J., Huang H., Song G., Huang K., Luo Y., Liu Q., He X., Cheng N. (2022). Intelligent biosensing strategies for rapid detection in food safety: A review. Biosens. Bioelectron..

[30] Kalita J.J., Sharma P., Bora U. (2022). Recent developments in application of nucleic acid aptamer in food safety. Food Control.

[31] Xia X., Yang H., Cao J., Zhang J., He Q., Deng R. (2022). Isothermal nucleic acid amplification for food safety analysis. TrAC Trends Anal. Chem..

[32] Kotsiri Z., Vidic J., Vantarakis A. (2022). Applications of biosensors for bacteria and virus detection in food and water-A systematic review. J. Environ. Sci..

[33] Zhang Z., Zhou J., Du X. (2019). Electrochemical Biosensors for Detection of Foodborne Pathogens. Micromachines.

[34] Ribeiro B.V., Ferreira L.F., Franco D.L. (2022). Advances in biosensor development for the determination of antibiotics in cow’s milk—A review. Talanta Open.

[35] Cao X., Chen C., Zhu Q. (2022). Biosensors based on functional nucleic acids and isothermal amplification techniques. Talanta.

[36] Zhao H., Lan X., Yu F., Li Z., Yang J., Du L. (2022). Comprehensive assessment of heavy metals in soil-crop system based on PMF and evolutionary game theory. Sci. Total Environ..

[37] Zhang X., Wu D., Zhou X., Yu Y., Liu J., Hu N., Wang H., Li G., Wu Y. (2019). Recent progress in the construction of nanozyme-based biosensors and their applications to food safety assay. TrAC Trends Anal. Chem..

[38] Penagos-Tabares F., Sulyok M., Faas J., Krska R., Khiaosa-Ard R., Zebeli Q. (2023). Residues of pesticides and veterinary drugs in diets of dairy cattle from conventional and organic farms in Austria. Environ. Pollut..

[39] Um S.H., Lee J.B., Park N., Kwon S.Y., Umbach C.C., Luo D. (2006). Enzyme-catalysed assembly of DNA hydrogel. Nat. Mater..

[40] Wang J., Chao J., Liu H., Su S., Wang L., Huang W., Willner I., Fan C. (2017). Clamped Hybridization Chain Reactions for the Self-Assembly of Patterned DNA Hydrogels. Angew. Chem. Int. Ed..

[41] Xiang B.B., He K.Y., Zhu R., Liu Z.L., Zeng S., Huang Y., Nie Z., Yao S.Z. (2016). Self-Assembled DNA Hydrogel Based on Enzymatically Polymerized DNA for Protein Encapsulation and Enzyme/DNAzyme Hybrid Cascade Reaction. ACS Appl. Mater. Interfaces.

[42] Sun Y., Li S., Chen R., Wu P., Liang J. (2020). Ultrasensitive and rapid detection of T-2 toxin using a target-responsive DNA hydrogel. Sens. Actuators B.

[43] Lu S.S., Shen J.L., Fan C.H., Li Q., Yang X.R. (2021). DNA Assembly-Based Stimuli-Responsive Systems. Adv. Sci..

[44] Ren J., Hu Y., Lu C.H., Guo W., Aleman-Garcia M.A., Ricci F., Willner I. (2015). pH-responsive and switchable triplex-based DNA hydrogels. Chem. Sci..

[45] Guo W., Qi X.J., Orbach R., Lu C.H., Freage L., Mironi-Harpaz I., Seliktar D., Yang H.H., Willner I. (2014). Reversible Ag(+)-crosslinked DNA hydrogels. Chem. Commun..

[46] Wu Y., Wang D., Willner I., Tian Y., Jiang L. (2018). Smart DNA Hydrogel Integrated Nanochannels with High Ion Flux and Adjustable Selective Ionic Transport. Angew. Chem. Int. Ed..

[47] Cangialosi A., Yoon C., Liu J., Huang Q., Guo J.K., Nguyen T.D., Gracias D.H., Schulman R. (2017). DNA sequence-directed shape change of photopatterned hydrogels via high-degree swelling. Science.

[48] Yao C., Tang H., Wu W., Tang J., Guo W., Luo D., Yang D. (2020). Double Rolling Circle Amplification Generates Physically Cross-Linked DNA Network for Stem Cell Fishing. J. Am. Chem. Soc..

[49] Gao X., Li X.Y., Sun X.Z., Zhang J.Y., Zhao Y.C., Liu X.J., Li F. (2020). DNA Tetrahedra-Cross-linked Hydrogel Functionalized Paper for Onsite Analysis of DNA Methyltransferase Activity Using a Personal Glucose Meter. Anal. Chem..

[50] Yu X., Hu Y.W., Kahn J.S., Cecconello A., Willner I. (2016). Orthogonal Dual-Triggered Shape-Memory DNA-Based Hydrogels. Chem. Eur. J..

[51] Chen F., He Y., Li Z., Xu B., Ye Q., Li X., Ma Z., Song W., Zhang Y. (2021). A novel tunable, highly biocompatible and injectable DNA-chitosan hybrid hydrogel fabricated by electrostatic interaction between chitosan and DNA backbone. Int. J. Pharm..

[52] Ko O., Han S., Lee J.B. (2020). Selective release of DNA nanostructures from DNA hydrogel. J. Ind. Eng. Chem..

[53] Tang J., Liang A., Yao C., Yang D. (2022). Assembly of Rolling Circle Amplification-Produced Ultralong Single-Stranded DNA to Construct Biofunctional DNA Materials. Chemistry.

[54] Xu W.L., Huang Y.S., Zhao H.R., Li P., Liu G.Y., Li J., Zhu C.S., Tian L.L. (2017). DNA Hydrogel with Tunable pH-Responsive Properties Produced by Rolling Circle Amplification. Chem. Eur. J..

[55] Bi S., Yue S., Zhang S. (2017). Hybridization chain reaction: A versatile molecular tool for biosensing, bioimaging, and biomedicine. Chem. Soc. Rev..

[56] Li Z.Y., Davidson-Rozenfeld G., Vazquez-Gonzalez M., Fadeev M., Zhang J.J., Tian H., Willner I. (2018). Multi-triggered Supramolecular DNA/Bipyridinium Dithienylethene Hydrogels Driven by Light, Redox, and Chemical Stimuli for Shape-Memory and Self-Healing Applications. J. Am. Chem. Soc..

[57] Alemdaroglu F.E., Herrmann A. (2007). DNA meets synthetic polymers—Highly versatile hybrid materials. Org. Biomol. Chem..

[58] Wang C., Fadeev M., Zhang J.J., Vazquez-Gonzalez M., Davidson-Rozenfeld G., Tian H., Willner I. (2018). Shape-memory and self-healing functions of DNA-based carboxymethyl cellulose hydrogels driven by chemical or light triggers. Chem. Sci..

[59] Fu X., Chen T., Song Y., Feng C., Chen H., Zhang Q., Chen G., Zhu X. (2021). mRNA Delivery by a pH-Responsive DNA Nano-Hydrogel. Small.

[60] Jeong J.Y., Do J.Y., Hong C.A. (2021). Target DNA- and pH-responsive DNA hydrogel-based capillary assay for the optical detection of short SARS-CoV-2 cDNA. Mikrochim. Acta.

[61] Hu Y., Gao S., Lu H., Ying J.Y. (2022). Acid-Resistant and Physiological pH-Responsive DNA Hydrogel Composed of A-Motif and i-Motif toward Oral Insulin Delivery. J. Am. Chem. Soc..

[62] Kang H., Liu H., Zhang X., Yan J., Zhu Z., Peng L., Yang H., Kim Y., Tan W. (2011). Photoresponsive DNA-cross-linked hydrogels for controllable release and cancer therapy. Langmuir.

[63] Zhang B., Wang C., Du Y., Paxton R., He X. (2021). A ‘smart’ aptamer-functionalized continuous label-free cell catch-transport-release system. J. Mater. Chem. B.

[64] Guo W.W., Lu C.H., Qi X.J., Orbach R., Fadeev M., Yang H.H., Willner I. (2014). Switchable Bifunctional Stimuli-Triggered Poly-N-Isopropylacrylamide/DNA Hydrogels. Angew. Chem. Int. Ed..

[65] Lin Y.N., Wang X.Y., Sun Y.L., Dai Y.X., Sun W.Y., Zhu X.D., Liu H., Han R., Gao D.D., Luo C.N. (2019). A chemiluminescent biosensor for ultrasensitive detection of adenosine based on target-responsive DNA hydrogel with Au@HKUST-1 encapsulation. Sens. Actuators B.

[66] Ma W., Liu M., Xie S., Liu B., Jiang L., Zhang X., Yuan X. (2022). CRISPR/Cas12a system responsive DNA hydrogel for label-free detection of non-glucose targets with a portable personal glucose meter. Anal. Chim. Acta.

[67] Zhang Z., Xie Z., Nie C., Wu S. (2022). Photo-controlled properties and functions of azobenzene-terminated polymers. Polymer.

[68] Simeth N.A., de Mendoza P., Dubach V.R.A., Stuart M.C.A., Smith J.W., Kudernac T., Browne W.R., Feringa B.L. (2022). Photoswitchable architecture transformation of a DNA-hybrid assembly at the microscopic and macroscopic scale. Chem. Sci..

[69] Huang F., Chen M., Zhou Z., Duan R., Xia F., Willner I. (2021). Spatiotemporal patterning of photoresponsive DNA-based hydrogels to tune local cell responses. Nat. Commun..

[70] Zhang C.-Y., Zhang N.-H. (2021). Size dependent correlation between structure and apparent stiffness of viral DNA during temperature variation. J. Mech. Phys. Solids.

[71] Zhu X., Wu J., Shao F., Hu X. (2018). Reversible Thermal Cycling of DNA Material for Efficient Cellulose Hydrolysis. ACS Appl. Bio Mater..

[72] Wang G., Wang S., Yan C., Bai G., Liu Y. (2018). DNA-functionalized gold nanoparticle-based fluorescence polarization for the sensitive detection of silver ions. Colloids Surf. B Biointerfaces.

[73] Xing X., Feng Y., Yu Z., Hidaka K., Liu F., Ono A., Sugiyama H., Endo M. (2019). Direct Observation of the Double-Stranded DNA Formation through Metal Ion-Mediated Base Pairing in the Nanoscale Structure. Chemistry.

[74] Liu X., Zhang J.J., Fadeev M., Li Z.Y., Wulf V., Tian H., Willner I. (2019). Chemical and photochemical DNA “gears” reversibly control stiffness, shape-memory, self-healing and controlled release properties of polyacrylamide hydrogels. Chem. Sci..

[75] Kahn J.S., Trifonov A., Cecconello A., Guo W.W., Fan C.H., Willner I. (2015). Integration of Switchable DNA-Based Hydrogels with Surfaces by the Hybridization Chain Reaction. Nano Lett..

[76] Hou M., Yin X., Jiang J.H., He J.J. (2021). DNAzyme-Triggered Sol-Gel-Sol Transition of a Hydrogel Allows Target Cell Enrichment. ACS Appl. Mater. Interfaces.

[77] Kahn J.S., Hu Y.W., Willner I. (2017). Stimuli-Responsive DNA-Based Hydrogels: From Basic Principles to Applications. Acc. Chem. Res..

[78] He Y., Yang X., Yuan R., Chai Y.Q. (2017). Switchable Target-Responsive 3D DNA Hydrogels As a Signal Amplification Strategy Combining with SERS Technique for Ultrasensitive Detection of miRNA 155. Anal. Chem..

[79] Bae S.W., Lee J.S., Harms V.M., Murphy W.L. (2019). Dynamic, Bioresponsive Hydrogels via Changes in DNA Aptamer Conformation. Macromol. Biosci..

[80] Hegde M., Pai P., Gangadhar Shetty M., Sundara Babitha K. (2022). Gold nanoparticle based biosensors for rapid pathogen detection: A Review. Environ. Nanotechnol. Monit. Manag..

[81] Jiang C., Wang F., Zhang K., Min T., Chen D., Wen Y. (2021). Distance-Based Biosensor for Ultrasensitive Detection of Uracil-DNA Glycosylase Using Membrane Filtration of DNA Hydrogel. ACS Sens..

[82] Wei X.F., Tian T., Jia S.S., Zhu Z., Ma Y.L., Sun J.J., Lin Z.Y., Yang C.J. (2015). Target-Responsive DNA Hydrogel Mediated “Stop-Flow” Microfluidic Paper-Based Analytic Device for Rapid, Portable and Visual Detection of Multiple Targets. Anal. Chem..

[83] Yanez-Aulestia A., Gupta N.K., Hernandez M., Osorio-Toribio G., Sanchez-Gonzalez E., Guzman-Vargas A., Rivera J.L., Ibarra I.A., Lima E. (2022). Gold nanoparticles: Current and upcoming biomedical applications in sensing, drug, and gene delivery. Chem. Commun..

[84] Guo Y., Zhao W. (2019). In situ formed nanomaterials for colorimetric and fluorescent sensing. Coord. Chem. Rev..

[85] Zhao M., Li Y., Ma X., Xia M., Zhang Y. (2019). Adsorption of cholesterol oxidase and entrapment of horseradish peroxidase in metal-organic frameworks for the colorimetric biosensing of cholesterol. Talanta.

[86] Fang B., Xu S., Huang Z., Wang S., Chen W., Yuan M., Hu S., Peng J., Lai W. (2020). Glucose oxidase-induced colorimetric immunoassay for qualitative detection of danofloxacin based on iron (Ⅱ) chelation reaction with phenanthroline. Food Chem..

[87] Gao L., Chen L., Zhang R., Yan X. (2022). Nanozymes: Next-generation artificial enzymes. Sci. Sin. Chim..

[88] Liu X., Huang D., Lai C., Qin L., Zeng G., Xu P., Li B., Yi H., Zhang M. (2019). Peroxidase-Like Activity of Smart Nanomaterials and Their Advanced Application in Colorimetric Glucose Biosensors. Small.

[89] Subhashree S., Kumar P.S. (2022). New analytical strategies amplified with carbon-based nanomaterial for sensing food pollutants. Chemosphere.

[90] Feng Y., Wang Y., Ying Y. (2021). Structural design of metal–organic frameworks with tunable colorimetric responses for visual sensing applications. Coord. Chem. Rev..

[91] Lan L., Yao Y., Ping J., Ying Y. (2017). Recent advances in nanomaterial-based biosensors for antibiotics detection. Biosens. Bioelectron..

[92] Chen M., Zhang J., Peng Y., Bai J., Li S., Han D., Ren S., Qin K., Zhou H., Han T. (2022). Design and synthesis of DNA hydrogel based on EXPAR and CRISPR/Cas14a for ultrasensitive detection of creatine kinase MB. Biosens. Bioelectron..

[93] Qiu F., Gan X., Yao J., Jiang B., Yuan R., Xiang Y. (2022). CRISPR/Cas12a-derived sensitive electrochemical biosensing of NF-kappaB p50 based on hybridization chain reaction and DNA hydrogel. Biosens. Bioelectron..

[94] Song H., Zhang Y., Wang S., Huang K., Luo Y., Zhang W., Xu W. (2020). Label-free polygonal-plate fluorescent-hydrogel biosensor for ultrasensitive microRNA detection. Sens. Actuators B.

[95] Yang Z.H., Zhuo Y., Yuan R., Chai Y.Q. (2017). Amplified impedimetric aptasensor combining target-induced DNA hydrogel formation with pH-stimulated signal amplification for the heparanase assay. Nanoscale.

[96] Han S., Dai R., Hu Y., Han L. (2022). Fluorometric and colorimetric detection of cerium(IV) ion using carbon dots and bathophenanthroline-disulfonate-ferrum(II) complex. Spectrochim. Acta A Mol. Biomol. Spectrosc..

[97] Lan L., Yao Y., Ping J., Ying Y. (2017). Recent Progress in Nanomaterial-Based Optical Aptamer Assay for the Detection of Food Chemical Contaminants. ACS Appl. Mater. Interfaces.

[98] Resch-Genger U., Grabolle M., Cavaliere-Jaricot S., Nitschke R., Nann T. (2008). Quantum dots versus organic dyes as fluorescent labels. Nat. Methods.

[99] Yang S., Li Y. (2020). Fluorescent hybrid silica nanoparticles and their biomedical applications. Wiley Interdiscip. Rev. Nanomed. Nanobiotechnol..

[100] Dong Y., Chen Z., Hou M., Qi L., Yan C., Lu X., Liu R., Xu Y. (2020). Mitochondria-targeted aggregation-induced emission active near infrared fluorescent probe for real-time imaging. Spectrochim. Acta A Mol. Biomol. Spectrosc..

[101] Farkkila S.M.A., Kiers E.T., Jaaniso R., Maeorg U., Leblanc R.M., Treseder K.K., Kang Z., Tedersoo L. (2021). Fluorescent nanoparticles as tools in ecology and physiology. Biol. Rev. Camb. Philos. Soc..

[102] Chang W.-H., Lee Y.-F., Liu Y.-W., Willner I., Liao W.-C. (2021). Stimuli-responsive hydrogel microcapsules for the amplified detection of microRNAs. Nanoscale.

[103] Xu K., Zhou R., Takei K., Hong M. (2019). Toward Flexible Surface-Enhanced Raman Scattering (SERS) Sensors for Point-of-Care Diagnostics. Adv. Sci..

[104] Chen H., Das A., Bi L., Choi N., Moon J.I., Wu Y., Park S., Choo J. (2020). Recent advances in surface-enhanced Raman scattering-based microdevices for point-of-care diagnosis of viruses and bacteria. Nanoscale.

[105] He Y., Yang X., Yuan R., Chai Y. (2019). A novel ratiometric SERS biosensor with one Raman probe for ultrasensitive microRNA detection based on DNA hydrogel amplification. J. Mater. Chem. B.

[106] Na W., Nam D., Lee H., Shin S. (2018). Rapid molecular diagnosis of infectious viruses in microfluidics using DNA hydrogel formation. Biosens. Bioelectron..

[107] Hong C.A., Park J.C., Na H., Jeon H., Nam Y.S. (2021). Short DNA-catalyzed formation of quantum dot-DNA hydrogel for enzyme-free femtomolar specific DNA assay. Biosens. Bioelectron..

[108] Chen Q., Tian R., Liu G., Wen Y., Bian X., Luan D., Wang H., Lai K., Yan J. (2022). Fishing unfunctionalized SERS tags with DNA hydrogel network generated by ligation-rolling circle amplification for simple and ultrasensitive detection of kanamycin. Biosens. Bioelectron..

[109] Cai W., Xie S.B., Zhang J., Tang D.Y., Tang Y. (2017). An electrochemical impedance biosensor for Hg2+ detection based on DNA hydrogel by coupling with DNAzyme-assisted target recycling and hybridization chain reaction. Biosens. Bioelectron..

[110] Hua Z., Yu T., Liu D., Xianyu Y. (2021). Recent advances in gold nanoparticles-based biosensors for food safety detection. Biosens. Bioelectron..

[111] Cheng W., Tang X., Zhang Y., Wu D., Yang W. (2021). Applications of metal-organic framework (MOF)-based sensors for food safety: Enhancing mechanisms and recent advances. Trends Food Sci. Technol..

[112] Qin G., Niu Z., Yu J., Li Z., Ma J., Xiang P. (2021). Soil heavy metal pollution and food safety in China: Effects, sources and removing technology. Chemosphere.

[113] Lu Y., Song S., Wang R., Liu Z., Meng J., Sweetman A.J., Jenkins A., Ferrier R.C., Li H., Luo W. (2015). Impacts of soil and water pollution on food safety and health risks in China. Environ. Int..

[114] Nilghaz A., Mousavi S.M., Li M., Tian J., Cao R., Wang X. (2021). Paper-based microfluidics for food safety and quality analysis. Trends Food Sci. Technol..

[115] Lin X., Wang Z., Jia X., Chen R., Qin Y., Bian Y., Sheng W., Li S., Gao Z. (2023). Stimulus-responsive hydrogels: A potent tool for biosensing in food safety. Trends Food Sci. Technol..

[116] Ye Y., Guo H., Sun X. (2019). Recent progress on cell-based biosensors for analysis of food safety and quality control. Biosens. Bioelectron..

[117] Cheng W., Wu X., Zhang Y., Wu D., Meng L., Chen Y., Tang X. (2022). Recent applications of hydrogels in food safety sensing: Role of hydrogels. Trends Food Sci. Technol..

[118] Yang Z., Chen L., McClements D.J., Qiu C., Li C., Zhang Z., Miao M., Tian Y., Zhu K., Jin Z. (2022). Stimulus-responsive hydrogels in food science: A review. Food Hydrocoll..

[119] Teng Y., Ni S., Wang J., Zuo R., Yang J. (2010). A geochemical survey of trace elements in agricultural and non-agricultural topsoil in Dexing area, China. J. Geochem. Explor..

[120] Zartman R.E. (2011). Treated Wastewater in Agriculture: Use and Impacts on the Soil Environment and Crops. J. Environ. Qual..

[121] Meng W., Wang Z., Hu B., Wang Z., Li H., Goodman R.C. (2016). Heavy metals in soil and plants after long-term sewage irrigation at Tianjin China: A case study assessment. Agric. Water Manag..

[122] Helwa Y., Dave N., Froidevaux R., Samadi A., Liu J.W. (2012). Aptamer-Functionalized Hydrogel Microparticles for Fast Visual Detection of Mercury(II) and Adenosine. ACS Appl. Mater. Interfaces.

[123] Huang Y.S., Ma Y.L., Chen Y.H., Wu X.M., Fang L.T., Zhu Z., Yang C.J. (2014). Target-Responsive DNAzyme Cross-Linked Hydrogel for Visual Quantitative Detection of Lead. Anal. Chem..

[124] Gao B.B., Liu H., Gu Z.Z. (2016). An exothermic chip for point-of-care testing using a forehead thermometer as a readout. Lab on a Chip.

[125] Kim H.S., Abbas N., Shin S. (2021). A rapid diagnosis of SARS-CoV-2 using DNA hydrogel formation on microfluidic pores. Biosens. Bioelectron..

[126] Zheng M., Liu H., Ye J., Ni B., Xie Y., Wang S. (2022). Target-responsive aptamer-cross-linked hydrogel sensors for the visual quantitative detection of aflatoxin B1 using exonuclease I-Triggered target cyclic amplification. Food Chemistry X.

[127] Huang Y.S., Fang L.T., Zhu Z., Ma Y.L., Zhou L.J., Chen X., Xu D.M., Yang C.Y. (2016). Design and synthesis of target-responsive hydrogel for portable visual quantitative detection of uranium with a microfluidic distance-based readout device. Biosens. Bioelectron..

[128] Nnachi R.C., Sui N., Ke B., Luo Z., Bhalla N., He D., Yang Z. (2022). Biosensors for rapid detection of bacterial pathogens in water, food and environment. Environ. Int..

[129] Ali A.A., Altemimi A.B., Alhelfi N., Ibrahim S.A. (2020). Application of Biosensors for Detection of Pathogenic Food Bacteria: A Review. Biosensors.

[130] Chen Y., Qian C., Liu C., Shen H., Wang Z., Ping J., Wu J., Chen H. (2020). Nucleic acid amplification free biosensors for pathogen detection. Biosens. Bioelectron..

[131] Wu H., Qian C., Wang R., Wu C., Wang Z., Wang L., Zhang M., Ye Z., Zhang F., He J.-S. (2020). Identification of pork in raw meat or cooked meatballs within 20 min using rapid PCR coupled with visual detection. Food Control.

[132] Cossettini A., Vidic J., Maifreni M., Marino M., Pinamonti D., Manzano M. (2022). Rapid detection of Listeria monocytogenes, Salmonella, Campylobacter spp., and Escherichia coli in food using biosensors. Food Control.

[133] Tariq L., Haagsma J., Havelaar A. (2011). Cost of illness and disease burden in The Netherlands due to infections with Shiga toxin-producing Escherichia coli O157. J. Food Prot..

[134] Zhang T., Tao Q., Bian X.-J., Chen Q., Yan J. (2021). Rapid Visualized Detection of Escherichia Coli O157:H7 by DNA Hydrogel Based on Rolling Circle Amplification. Chin. J. Anal. Chem..

[135] Yu J., Xiao S., Yu Z., Hui Y., Li T., Wu D., Bi W., Gan N., Jia Z. (2022). On-site and dual-mode detection of live Vibrio parahaemolyticus in waters: A universal pathogen sensing platform based on a smart hydrogel aptasensor imbedded with gold nanoclusters. Sens. Actuators B.

[136] Lee H.Y., Jeong H., Jung I.Y., Jang B., Seo Y.C., Lee H., Lee H. (2015). DhITACT: DNA Hydrogel Formation by Isothermal Amplification of Complementary Target in Fluidic Channels. Adv. Mater..

[137] Chen X., Xie Y.X., Zhang Y.Z., Li C.W., Xu W.T. (2020). Programmable 3D rigid clathrate hydrogels based on self-assembly of tetrahedral DNA and linker PCR products. Chem. Commun..

[138] Su W., Liang D., Tan M. (2021). Nucleic acid-based detection for foodborne virus utilizing microfluidic systems. Trends Food Sci. Technol..

[139] Nam J., Jang W.S., Kim J., Lee H., Lim C.S. (2019). Lamb wave-based molecular diagnosis using DNA hydrogel formation by rolling circle amplification (RCA) process. Biosens. Bioelectron..

[140] Wang R., Li Y. (2013). Hydrogel based QCM aptasensor for detection of avian influenza virus. Biosens. Bioelectron..

[141] Xu L., Wang R., Kelso L.C., Ying Y., Li Y. (2016). A target-responsive and size-dependent hydrogel aptasensor embedded with QD fluorescent reporters for rapid detection of avian influenza virus H5N1. Sens. Actuators B.

[142] Han S., Roy P.K., Hossain M.I., Byun K.H., Choi C., Ha S.D. (2021). COVID-19 pandemic crisis and food safety: Implications and inactivation strategies. Trends Food Sci. Technol..

[143] Yang T., Zhang Z., Zhao B., Hou R., Kinchla A., Clark J.M., He L. (2016). Real-Time and in Situ Monitoring of Pesticide Penetration in Edible Leaves by Surface-Enhanced Raman Scattering Mapping. Anal. Chem..

[144] Yang L., Zhao J., Wang C., Wang Z., Xing C., Guo H., Wang Y., Zhao Z., Hu Z., Cai Z. (2021). Bi/BiVO4/NiFe-LDH heterostructures with enhanced photoelectrochemical performance for streptomycin detection. J. Environ. Sci..

[145] Wang Z., Sun Y., Liang D., Zeng Y., He S., Mari G.M., Peng T., Jiang H. (2020). Highly sensitive chromatographic time-resolved fluoroimmunoassay for rapid onsite detection of streptomycin in milk. J. Dairy Sci..

[146] Du B., Wen F., Zhang Y., Zheng N., Li S., Li F., Wang J. (2019). Presence of tetracyclines, quinolones, lincomycin and streptomycin in milk. Food Control.

[147] Luo Y., Tan X., Young D.J., Chen Q., Huang Y., Feng D., Ai C., Mi Y. (2020). A photoelectrochemical aptasensor for the sensitive detection of streptomycin based on a TiO(2)/BiOI/BiOBr heterostructure. Anal. Chim. Acta.

[148] Wang X., Chen C., Waterhouse G.I.N., Qiao X., Xu Z. (2022). Ultra-sensitive detection of streptomycin in foods using a novel SERS switch sensor fabricated by AuNRs array and DNA hydrogel embedded with DNAzyme. Food Chem..

[149] Deng J., Liu Y., Lin X., Lyu Y., Qian P., Wang S. (2018). A ratiometric fluorescent biosensor based on cascaded amplification strategy for ultrasensitive detection of kanamycin. Sens. Actuators B.

[150] Qin L., Zeng G., Lai C., Huang D., Zhang C., Xu P., Hu T., Liu X., Cheng M., Liu Y. (2017). A visual application of gold nanoparticles: Simple, reliable and sensitive detection of kanamycin based on hydrogen-bonding recognition. Sens. Actuators B.

[151] Tan B., Zhao H., Du L., Gan X., Quan X. (2016). A versatile fluorescent biosensor based on target-responsive graphene oxide hydrogel for antibiotic detection. Biosens. Bioelectron..

[152] Tang J., Liu L., Gao S., Qin J., Liu X., Tang D. (2021). A portable thermal detection method based on the target responsive hydrogel mediated self-heating of a warming pad. Chem. Commun..

[153] Jigyasa, Rajput J.K. (2022). Nanomaterial-based sensors as potential remedy for detection of biotoxins. Food Control.

[154] Shan H., Li X., Liu L., Song D., Wang Z. (2020). Recent advances in nanocomposite-based electrochemical aptasensors for the detection of toxins. J. Mater. Chem. B.

[155] Ji B., Kenaan A., Gao S., Cheng J., Cui D., Yang H., Wang J., Song J. (2019). Label-free detection of biotoxins via a photo-induced force infrared spectrum at the single-molecular level. Analyst.

[156] Tang L.Y., Huang Y.Y., Lin C.Y., Qiu B., Guo L.H., Luo F., Lin Z.Y. (2020). Highly sensitive and selective aflatoxin B-1 biosensor based on Exonuclease I-catalyzed target recycling amplification and targeted response aptamer-crosslinked hydrogel using electronic balances as a readout. Talanta.

[157] Xie Y., Wang W., Zhang S. (2019). Purification and identification of an aflatoxin B1 degradation enzyme from Pantoea sp. T6. Toxicon.

[158] Ma Y., Mao Y., Huang D., He Z., Yan J., Tian T., Shi Y., Song Y., Li X., Zhu Z. (2016). Portable visual quantitative detection of aflatoxin B1 using a target-responsive hydrogel and a distance-readout microfluidic chip. Lab Chip.

[159] Zhao M.M., Wang P.L., Guo Y.J., Wang L.X., Luo F., Qiu B., Guo L.H., Su X.O., Lin Z.Y., Chen G.N. (2018). Detection of aflatoxin B-1 in food samples based on target-responsive aptamer-cross-linked hydrogel using a handheld pH meter as readout. Talanta.

[160] Quintela S., Villarán M.C., López de Armentia I., Elejalde E. (2013). Ochratoxin A removal in wine: A review. Food Control.

[161] Jiang C., Lan L., Yao Y., Zhao F., Ping J. (2018). Recent progress in application of nanomaterial-enabled biosensors for ochratoxin A detection. TrAC Trends Anal. Chem..

[162] Liu R.D., Huang Y.S., Ma Y.L., Jia S.S., Gao M.X., Li J.X., Zhang H.M., Xu D.M., Wu M., Chen Y. (2015). Design and Synthesis of Target-Responsive Aptamer-Cross-linked Hydrogel for Visual Quantitative Detection of Ochratoxin A. ACS Appl. Mater. Interfaces.

[163] Hao L.L., Wang W., Shen X.Q., Wang S.L., Li Q., An F.L., Wu S.J. (2020). A Fluorescent DNA Hydrogel Aptasensor Based on the Self-Assembly of Rolling Circle Amplification Products for Sensitive Detection of Ochratoxin A. J. Agric. Food Chem..

[164] Hao W., Ge Y., Qu M., Wen Y., Liang H., Li M., Chen C., Xu L. (2022). A simple rapid portable immunoassay of trace zearalenone in feed ingredients and agricultural food. J. Food Compos. Anal..

[165] Sun Y., Qi S., Dong X., Qin M., Zhang Y., Wang Z. (2022). Colorimetric aptasensor targeting zearalenone developed based on the hyaluronic Acid-DNA hydrogel and bimetallic MOFzyme. Biosens. Bioelectron..

[166] Sun Y., Lv Y., Zhang Y., Wang Z. (2022). A Stimuli-Responsive Colorimetric Aptasensor Based on the DNA Hydrogel-Coated MOF for Fumonisin B1 Determination in Food Samples. Food Chem..

[167] Wu L., Zhang C., Long Y., Chen Q., Zhang W., Liu G. (2022). Food additives: From functions to analytical methods. Crit. Rev. Food Sci. Nutr..

[168] Li J., Gao X., He Y., Wang L., Wang Y., Zeng L. (2022). Elevated emissions of melamine and its derivatives in the indoor environments of typical e-waste recycling facilities and adjacent communities and implications for human exposure. J. Hazard. Mater..

[169] Wang Z., Chen R., Hou Y., Qin Y., Li S., Yang S., Gao Z. (2022). DNA hydrogels combined with microfluidic chips for melamine detection. Anal. Chim. Acta.

[170] Liu D., Jia S.S., Zhang H.M., Ma Y.L., Guan Z.C., Li J.X., Zhu Z., Ji T.H., Yang C.J. (2017). Integrating Target-Responsive Hydrogel with Pressuremeter Readout Enables Simple, Sensitive, User-Friendly, Quantitative Point-of-Care Testing. ACS Appl. Mater. Interfaces.

[171] Li Y., Ma Y., Jiao X., Li T., Lv Z., Yang C.J., Zhang X., Wen Y. (2019). Control of capillary behavior through target-responsive hydrogel permeability alteration for sensitive visual quantitative detection. Nat. Commun..

[172] Lim H.J., Lee E.H., Lee S.D., Yoon Y., Son A. (2018). Quantitative screening for endocrine-disrupting bisphenol A in consumer and household products using NanoAptamer assay. Chemosphere.

[173] Wang J.Y., Guo Q.Y., Yao Z.Y., Yin N., Ren S.Y., Li Y., Li S., Peng Y., Bai J.L., Ning B.A. (2020). A low-field nuclear magnetic resonance DNA-hydrogel nanoprobe for bisphenol A determination in drinking water. Mikrochim. Acta.

[174] Wu H., Zhang X., Wu B., Qian C., Zhang F., Wang L., Ye Z., Wu J. (2020). Rapid on-site detection of genetically modified soybean products by real-time loop-mediated isothermal amplification coupled with a designed portable amplifier. Food Chem..

[175] Zeng H., Wang J., Jia J., Wu G., Yang Q., Liu X., Tang X. (2021). Development of a lateral flow test strip for simultaneous detection of BT-Cry1Ab, BT-Cry1Ac and CP4 EPSPS proteins in genetically modified crops. Food Chem..

[176] Cheng N., Shang Y., Xu Y., Zhang L., Luo Y., Huang K., Xu W. (2017). On-site detection of stacked genetically modified soybean based on event-specific TM-LAMP and a DNAzyme-lateral flow biosensor. Biosens. Bioelectron..

[177] Gryadunov D.A., Getman I.A., Chizhova S.I., Mikhailovich V.M., Zasedatelev A.S., Romanov G.A. (2011). Identification of plant-derived genetically modified organisms in food and feed using a hydrogel oligonucleotide microchip. Mol. Biol..

[178] Bucur B., Purcarea C., Andreescu S., Vasilescu A. (2021). Addressing the Selectivity of Enzyme Biosensors: Solutions and Perspectives. Sensors.

[179] Wang Q., Zhao Y., Yang Q., Du D., Yang H., Lin Y. (2019). Amperometric sarcosine biosensor with strong anti-interference capabilities based on mesoporous organic-inorganic hybrid materials. Biosens. Bioelectron..

[180] Ayenimo J.G., Adeloju S.B. (2017). Amperometric detection of glucose in fruit juices with polypyrrole-based biosensor with an integrated permselective layer for exclusion of interferences. Food Chem..

[181] Mao X.X., Chen G.F., Wang Z.H., Zhang Y.G., Zhu X.L., Li G.X. (2018). Surface-immobilized and self-shaped DNA hydrogels and their application in biosensing. Chem. Sci..

[182] Pi K., Liu J., Van Cappellen P. (2020). A DNA-based biosensor for aqueous Hg(II): Performance under variable pH, temperature and competing ligand composition. J. Hazard. Mater..

